# siRNAs containing 2′-fluorinated *Northern*-methanocarbacyclic (2′-F-NMC) nucleotides: *in vitro* and *in vivo* RNAi activity and inability of mitochondrial polymerases to incorporate 2′-F-NMC NTPs

**DOI:** 10.1093/nar/gkab050

**Published:** 2021-02-12

**Authors:** Masaaki Akabane-Nakata, Namrata D Erande, Pawan Kumar, Rohan Degaonkar, Jason A Gilbert, June Qin, Martha Mendez, Lauren Blair Woods, Yongfeng Jiang, Maja M Janas, Derek K O’Flaherty, Ivan Zlatev, Mark K Schlegel, Shigeo Matsuda, Martin Egli, Muthiah Manoharan

**Affiliations:** Alnylam Pharmaceuticals, 675 West Kendall Street, Cambridge, MA 02142, USA; Alnylam Pharmaceuticals, 675 West Kendall Street, Cambridge, MA 02142, USA; Alnylam Pharmaceuticals, 675 West Kendall Street, Cambridge, MA 02142, USA; Alnylam Pharmaceuticals, 675 West Kendall Street, Cambridge, MA 02142, USA; Alnylam Pharmaceuticals, 675 West Kendall Street, Cambridge, MA 02142, USA; Alnylam Pharmaceuticals, 675 West Kendall Street, Cambridge, MA 02142, USA; Alnylam Pharmaceuticals, 675 West Kendall Street, Cambridge, MA 02142, USA; Alnylam Pharmaceuticals, 675 West Kendall Street, Cambridge, MA 02142, USA; Alnylam Pharmaceuticals, 675 West Kendall Street, Cambridge, MA 02142, USA; Alnylam Pharmaceuticals, 675 West Kendall Street, Cambridge, MA 02142, USA; Alnylam Pharmaceuticals, 675 West Kendall Street, Cambridge, MA 02142, USA; Alnylam Pharmaceuticals, 675 West Kendall Street, Cambridge, MA 02142, USA; Alnylam Pharmaceuticals, 675 West Kendall Street, Cambridge, MA 02142, USA; Alnylam Pharmaceuticals, 675 West Kendall Street, Cambridge, MA 02142, USA; Department of Biochemistry, School of Medicine, Vanderbilt University, Nashville, TN 37232, USA; Alnylam Pharmaceuticals, 675 West Kendall Street, Cambridge, MA 02142, USA

## Abstract

We recently reported the synthesis of 2′-fluorinated *Northern*-methanocarbacyclic (2′-F-NMC) nucleotides, which are based on a bicyclo[3.1.0]hexane scaffold. Here, we analyzed RNAi-mediated gene silencing activity in cell culture and demonstrated that a single incorporation of 2′-F-NMC within the guide or passenger strand of the tri-*N*-acetylgalactosamine-conjugated siRNA targeting mouse *Ttr* was generally well tolerated. Exceptions were incorporation of 2′-F-NMC into the guide strand at positions 1 and 2, which resulted in a loss of the *in vitro* activity. Activity at position 1 was recovered when the guide strand was modified with a 5′ phosphate, suggesting that the 2′-F-NMC is a poor substrate for 5′ kinases. In mice, the 2′-F-NMC-modified siRNAs had comparable RNAi potencies to the parent siRNA. 2′-F-NMC residues in the guide seed region position 7 and at positions 10, 11 and 12 were well tolerated. Surprisingly, when the 5′-phosphate mimic 5′-(*E*)-vinylphosphonate was attached to the 2′-F-NMC at the position 1 of the guide strand, activity was considerably reduced. The steric constraints of the bicyclic 2′-F-NMC may impair formation of hydrogen-bonding interactions between the vinylphosphonate and the MID domain of Ago2. Molecular modeling studies explain the position- and conformation-dependent RNAi-mediated gene silencing activity of 2′-F-NMC. Finally, the 5′-triphosphate of 2′-F-NMC is not a substrate for mitochondrial RNA and DNA polymerases, indicating that metabolites should not be toxic.

## INTRODUCTION

**Figure 1.**
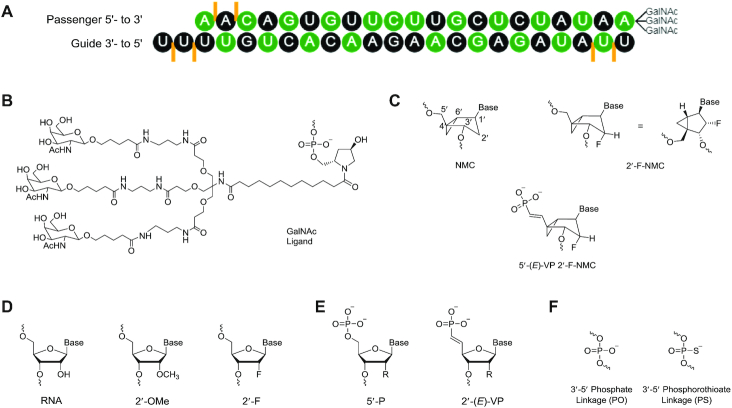
(**A**) Sequence overview of the parent siRNA-GalNAc conjugate duplex targeting mouse *Ttr* used in this study. This siRNA was previously characterized (18). Black: 2′-*O*-methyl (2′-OMe), green: 2′-deoxy-2′-fluoro (2′-F), orange lines: phosphorothioate linkage. (**B**) Structure of GalNAc ligand (**C**) Structures of *northern* methanocarbacyclic (NMC), 2′-F-NMC monomers, and 5′-(*E*)-vinylphosphonate (5′-(*E*)-VP) 2′-F-NMC used in this study. (**D**) Structures of the other nucleotides mentioned in this study, including 2′-*O*-methyl (2′-OMe), 2′-deoxy-2′-fluoro (2′-F). (**E**) Structures of the other 5′-end modifications mentioned in this study, including 5′-monophosphate (5′-P), 5′-(*E*)-vinylphosphonate (5′-(*E*)-VP), with R = OCH_3_ (2′-OMe), or OCH_2_CONHCH_3_ (2′-*O*-NMA) (**F**) Structures of backbone modifications include both natural phosphate (PO) and phosphorothioate (PS).

Therapeutics that act through the RNA interference (RNAi) pathway prevent production of disease-causing proteins (1–3). Synthetic small interfering RNAs (siRNAs), which induce gene silencing via the endogenous RNAi process, are chemically modified to increase stability against nuclease degradation, to facilitate their cellular uptake through cell-membrane, and to reduce their immune stimulation (4,5). The first RNAi therapeutic to be approved for clinical use was patisiran (ONPATTRO^®^), which is used to treat patients with polyneuropathy caused by hereditary ATTR amyloidosis. This siRNA is partially modified with 2′-*O*-methyl (2′-OMe) and encapsulated in lipid nanoparticles (6). A second RNAi therapeutic, givosiran (GIVLAARI^®^), (https://www.fda.gov/drugs/resources-information-approved-drugs/fda-approves-givosiran-acute-hepatic-porphyria), has been approved for the treatment of acute hepatic porphyrias (7,8). More recently, both US FDA and EMA have approved a third RNAi drug, lumasiran (OXLUMO^®^), (https://www.fda.gov/news-events/press-announcements/fda-approves-first-drug-treat-rare-metabolic-disorder), for the treatment of primary hyperoxaluria type 1 in all age groups (9), and EMA has approved the fourth RNAi drug, inclisiran (Leqvio^®^), (https://www.novartis.com/news/media-releases/novartis-receives-eu-approval-leqvio-inclisiran-first-class-sirna-lower-cholesterol-two-doses-year), for the treatment of adults with heterozygous familial hypercholesterolemia (10–12). These latter three compounds are fully modified with 2′-deoxy-2′-fluoro (2′-F) and 2′-*O*-methyl (2′-OMe) (Figure 1) to confer stability in the absence of a lipid. These modifications are tolerated by the endonuclease silencer enzyme Argonaute-2 (Ago2), the catalytic component of the RNA-induced silencing complex (RISC) (13–17). The passenger strands of these siRNAs are conjugated with a tri-*N*-acetylgalactosamine (GalNAc; Figure 1B), which interacts with the asialoglycoprotein receptor that is highly expressed on hepatocytes, to facilitate specific delivery into the liver (8,9,12,18).

The 2′-F residues adopt a C3′-*endo* or *North* sugar conformation because of the gauche interaction between F2′ and O4′, thereby reducing the entropic penalty for formation of the A-form duplex. In addition to the entropic stabilization, the electron-withdrawing power of the 2′-F substituent enhances Watson–Crick hydrogen-bonding and base stacking interactions, the main contributors to the enthalpic stabilization (16). Furthermore, we found that 2′-F-modified siRNAs have reduced immune stimulation and improved activity *in vitro* and *in vivo* compared with unmodified RNA (14). However, oligonucleotides containing 2′-F residues are more sensitive to nuclease-mediated degradation than those containing other 2′-modified nucleotides such as 2′-OMe, and 2′-F monomers are recognized, albeit poorly, by human RNA polymerases at high concentrations (19,20). Hence, introduction of a fluorine substituent into other sugar modifications is an attractive approach to retain the advantages while overcoming limitations of 2′-F-RNA (21–37).

**Figure 2.**
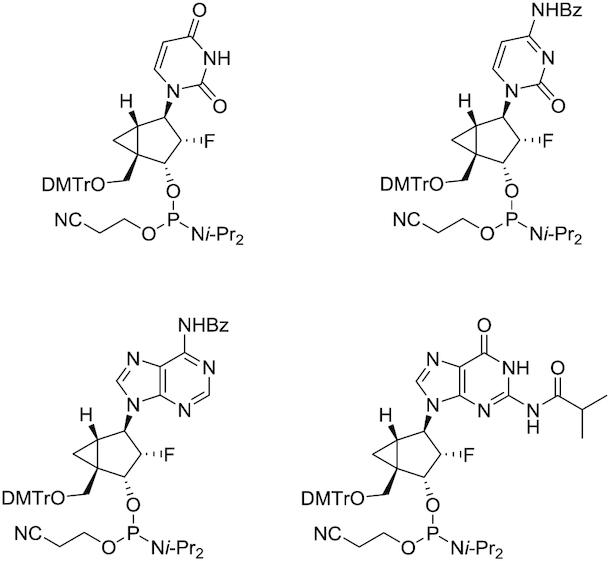
Structure of 2′-F-NMC phosphoramidites bearing all four RNA nucleobases.

*Northern* methanocarbacyclic (NMC) nucleosides are based on a carbocyclic bicyclo[3.1.0]hexane system (Figure 1C), and have been recently reviewed due to the polypharmacological interest (38). The bicyclic NMC sugar, discovered in 1990s independently by Altmann and Marquez research groups (39, 40), adopts the pseudoboat C2′-*exo* (*North*) conformation and is an effective structural mimic of the C3′-*endo* conformation observed in RNA duplexes due to the methylene bridge between the C4′ and C6′ positions. Consequently, NMC-modified oligonucleotides form stable duplexes with RNA and enhance resistance to nuclease degradation (39–44). In the only crystal structure of an oligonucleotide bearing NMC residues determined at high resolution (1.8 Å), the modified residues had a C4′-*exo* pucker (44). It is possible that this conformation is a consequence of the location of the NMC residues in a loop, however. Jung *et al.* synthesized 2′-fluorinated NMC (2′-F-NMC; Figure 1C) thymidine analogs and oligonucleotides containing this building block (25). Deoxyoligonucleotides containing 2′-F-NMC residues have higher affinity for RNA than those containing 2′-F uridine or NMC thymidine (26). This stabilization encouraged us to explore the potential of the 2′-F-NMC scaffold as a chemical modification in RNAi-based therapeutics. To investigate the sequence- and position-dependent RNAi activity, we recently synthesized 2′-F-NMC analogs bearing all four RNA nucleobases (A, U, G, C) and oligonucleotides containing these analogs (Figure 2) and described the binding affinities to a target RNA and reduced susceptibilities to exonuclease of these oligonucleotides (37).

In the present study, we evaluated the *in vitro* and *in vivo* gene silencing potency and off-target activity of 2′-F-NMC-modified siRNA. Our laboratory has demonstrated that the impact of chemically modified nucleotides on siRNA pharmacology strongly depends on both sequence and position in an siRNA (17), therefore we explored the effects of a single 2′-F-NMC at all positions of the guide and passenger strands of an siRNA. In addition, we describe synthesis of the 2′-F-NMC uridine modified with 5′-(*E*)-vinylphosphonate (5′-(*E*)-VP; Figure 1E), a metabolically stable 5′-monophosphate mimic, and effects of this residue on gene silencing activity when placed at the 5′ end of the guide strand of the siRNA. In the RNAi pathway, 5′-end phosphorylation of the guide strand by natural kinases enhances loading into the Ago2 enzyme due to hydrogen-bonding interactions between the 5′-monophosphate of the guide strand and the MID domain of Ago2 (45–47). However, 5′-phosphorylation of the guide strand is ineffective *in vivo*, likely due to poor substrate nature of the chemically modified siRNAs for kinases and vulnerability of the resulting monophosphate group to phosphatases (48–50). We and other groups have previously reported that a 5′-(*E*)-VP, in combination with 2′-modified nucleotides, enhances RISC loading and RNAi efficacy compared to the non-phosphorylated siRNA (49–54). To the best of our knowledge, the 5′-(*E*)-VP modification with conformationally restricted nucleic acids bearing bicyclic or tricyclic scaffolds has never been reported for RNAi-mediated therapeutics. Therefore, we evaluated potency of an siRNA with the 5′-(*E*)-VP 2′-F-NMC on the 5′ end of the guide strand. We also evaluated the seed region-mediated off-target modulation potential of an siRNA modified with 2′-F-NMC in the guide strand. Finally, we synthesized pyrimidine 2′-F-NMC nucleoside 5′-triphosphates (NTPs) and evaluated the efficiency with which these are used as substrates by the mitochondrial RNA polymerase POLRMT and the mitochondrial DNA polymerase POLG and compared it against RNA NTPs, DNA NTPs and 2′-F NTPs to predict the safety properties of this new modification.

## MATERIALS AND METHODS

### Synthetic procedures and compound characterization

#### General conditions

TLC was performed on Merck silica gel 60 plates coated with F254. Compounds were visualized under UV light (254 nm) or after spraying with the *p*-anisaldehyde staining solution followed by heating. Flash column chromatography was performed using a Teledyne ISCO Combi Flash system with pre-packed RediSep Teledyne ISCO silica gel cartridges. All moisture-sensitive reactions were carried out under anhydrous conditions using dry glassware, anhydrous solvents, and argon atmosphere. All commercially available reagents and solvents were purchased from Sigma-Aldrich unless otherwise stated and were used as received. ESI-MS spectra were recorded on a Waters Qtof Premier instrument using the direct flow injection mode. ^1^H NMR spectra were recorded at 400 or 500 MHz. ^13^C NMR spectra were recorded at 101 or 126 MHz. ^19^F NMR spectra were recorded at 470 MHz. ^31^P NMR spectra were recorded at 202 MHz. Chemical shifts are given in ppm referenced to the solvent residual peak (DMSO-*d*_6_ – ^1^H: δ at 2.50 ppm and ^13^C δ at 39.5 ppm; CD_3_CN – ^1^H: δ at 1.94 ppm and ^13^C δ at 1.32 and 118.3 ppm; D_2_O – ^1^H: δ at 4.79 ppm). Coupling constants are given in Hertz. Signal splitting patterns are described as singlet (s), doublet (d), triplet (t), broad signal (brs), or multiplet (m).

#### Synthesis of compound 2

Compound **1** (37) (5 g, 7.43 mmol) was dissolved in 80% AcOH (aq.) (45 ml). The mixture was stirred at rt for 48 h. The mixture was diluted with ethyl acetate and washed with saturated NaHCO_3_ (aq.) and brine. The organic layer was dried over Na_2_SO_4_ and concentrated under vacuum. The crude residue was purified by column chromatography on silica gel (50–100% ethyl acetate in hexane) to give compound **2** as a white solid (1.83 g, 67%). ^1^H NMR (500 MHz, DMSO-*d*_6_) δ 11.30 (s, 1H), 7.87 (d, *J* = 8.0 Hz, 1H), 5.58 (d, *J* = 8.0 Hz, 1H), 5.02 (dd, *J* = 4.6 and 4.6 Hz, 1H), 4.74 (d, *J* = 17.4 Hz, 1H), 4.65 (dd, *J* = 5.6 and 9.7 Hz, 1H), 4.58 (d, *J* = 5.6 and 23.4 Hz, 1H), 3.94 (dd, *J* = 4.6 and 11.3 Hz, 1H), 3.12 (d, *J* = 4.6 and 11.3 Hz, 1H), 1.34 (dd, *J* = 3.8 and 8.9 Hz, 1H), 0.94 (dd, *J* = 3.8 and 3.8 Hz, 1H), 0.86 (s, 9H), 0.70–0.73 (m, 1H), 0.08 (s, 3H), 0.04 (s, 3H). ^13^C NMR (126 MHz, DMSO-*d*_6_) δ 163.2, 150.8, 141.3, 101.5, 94.5 (d, *J* = 194.0 Hz), 71.0 (d, *J* = 16.4 Hz), 61.2, 60.2 (d, *J* = 26.5 Hz), 36.6, 25.6, 20.3, 17.9, 10.7 (d, *J* = 7.6 Hz), –4.95, –5.07. ^19^F NMR (470 MHz, DMSO-*d*_6_) δ –186.82–186.63 (m). HRMS calc. for C_17_H_28_FN_2_O_4_Si [M+H]^+^ 371.1797, found 371.1803.

#### Synthesis of compound 3

To a solution of **2** (60 mg, 0.162 mmol) in CH_2_Cl_2_ (1.6 ml) and pyridine (0.4 ml) was added Dess-Martin periodinane (137 mg, 0.324 mmol) at 0°C, and the mixture was stirred at 0°C for 3 h. The reaction mixture was quenched with saturated Na_2_S_2_O_3_ (aq.) and washed with saturated NaHCO_3_ (aq.) and water. The organic layer was concentrated under vacuum. The crude residue was purified by column chromatography on silica gel (75–100% ethyl acetate in hexane) to give compound **3** as a white solid (59.1 mg, 99%). ^1^H NMR (500 MHz, DMSO-*d*_6_) δ 11.33 (s, 1H), 8.90 (s, 1H), 7.58 (d, *J* = 8.0 Hz, 1H), 5.60 (d, *J* = 8.0 Hz, 1H), 5.02 (dd, *J* = 6.1 and 10.5 Hz, 1H), 4.94 (ddd, *J* = 2.7, 6.1 and 50.0 Hz, 1H), 4.61 (dd, *J* = 2.7 and 17.8 Hz, 1H), 2.20–2.23 (m, 1H), 1.74–1.77 (m, 1H), 1.50 (dd, *J* = 5.6 and 5.6 Hz, 1H), 0.85 (s, 9H), 0.07 (s, 6H). ^13^C NMR (126 MHz, DMSO-*d*_6_) δ 198.2, 163.3, 150.8, 143.1, 101.7, 94.9 (d, *J* = 196.6 Hz), 67.5 (d, *J* = 16.4 Hz), 62.5 (d, *J* = 26.5 Hz), 46.1, 26.8 (d, *J* = 7.6 Hz), 25.6, 17.9, 16.2 (d, *J* = 3.8 Hz), -5.00, -5.06. ^19^F NMR (470 MHz, DMSO-*d*_6_) δ –186.47 to –186.30 (m). HRMS calc. for C_17_H_26_FN_2_O_4_Si [M+H]^+^ 369.1640, found 369.1650.

#### Synthesis of compound 4

To a suspension of sodium hydride (60% in mineral oil, 217 mg, 5.43 mmol) in THF (10 ml) was added dropwise a solution of tetra(pivaloyloxymethyl)-bis-phosphonate **7** (53) (3.43 g, 5.43 mmol) in THF (10 ml) at −78°C. The mixture was stirred at −78°C for 30 min. A solution of **3** (1 g, 2.71 mmol) in THF (7 ml) was added dropwise at −78°C, and the mixture was stirred at rt. After 15 h, the reaction was quenched by addition of saturated NH_4_Cl (aq.). The reaction mixture was extracted with CH_2_Cl_2_ and ethyl acetate. The combined organic layer was washed with brine, dried (Na_2_SO_4_) and concentrated under vacuum. The crude residue was purified by column chromatography on silica gel (75–100% ethyl acetate in hexanes) to obtain compound **4** as a white foam (1.71 g, 93.4%). ^1^H NMR (400 MHz, DMSO-*d*_6_) δ 11.33 (s, 1H), 7.36 (d, *J* = 8.0 Hz, 1H), 6.73 (dd, *J* = 17.3 and 23.8 Hz, 1H), 5.83 (dd, *J* = 17.3 and 20.4 Hz, 1H), 5.55–5.59 (m, 5H), 4.91 (dd, *J* = 6.1 and 10.7 Hz, 1H), 4.83 (dd, *J* = 6.1 and 26.3 Hz, 1H), 4.68 (d, *J* = 19.2 Hz, 1H), 1.82–1.86 (m, 1H), 1.30–1.32 (m, 2H), 1.15 (s, 9H), 1.14 (s, 9H), 0.84 (s, 9H), 0.09 (s, 3H), 0.05 (s, 3H). ^13^C NMR (101 MHz, DMSO-*d*_6_) δ 176.0, 175.9, 163.2, 154.6 (d, *J* = 7.1 Hz), 150.7, 142.4, 112.8 (d, *J* = 192.9 Hz), 101.7, 94.6 (d, *J* = 193.9 Hz), 81.3 (d, *J* = 6.1 Hz), 81.3 (d, *J* = 6.1 Hz), 72.6 (d, *J* = 16.2 Hz), 62.6 (d, *J* = 27.3 Hz), 38.2 (d, *J* = 14.1 Hz), 38.1 (d, *J* = 11.1 Hz), 28.0, 26.4, 25.6, 17.7, 16.1 (d, *J* = 4.0 Hz), –4.70, –5.05. ^19^F NMR (470 MHz, DMSO-*d*_6_) δ –186.06 to –185.88 (m). ^31^P NMR (202 MHz, DMSO-*d*_6_) δ 19.83. HRMS calc. for C_30_H_48_FN_2_NaO_10_PSi [M+Na]^+^ 697.2692, found 697.2698.

#### Synthesis of compound 5

A solution of **4** (1.7 g, 2.52 mmol) in HCO_2_H/H_2_O (1:1) (26 ml) was stirred at rt for 24 h. The reaction mixture was concentrated under vacuum. The crude residue was purified by column chromatography on silica gel (80–100% ethyl acetate in hexane) to give compound **5** as a white foam (1.31 g, 93%). ^1^H NMR (400 MHz, DMSO-*d*_6_) δ 11.34 (s, 1H), 7.25 (d, *J* = 8.0 Hz, 1H), 6.63 (dd, *J* = 17.3 and 23.9 Hz, 1H), 5.97 (dd, *J* = 17.3 and 21.0 Hz, 1H), 5.55–5.62 (m, 5H), 5.25 (brs, 1H), 4.64–4.82 (m, 3H), 1.86 (dd, *J* = 4.6 and 8.6 Hz, 1H),1.37–1.39 (m, 1H), 1.15–1.26 (m, 19H). ^13^C NMR (126 MHz, DMSO-*d*_6_) δ 176.1, 163.2, 155.0 (d, *J* = 6.3 Hz), 150.6, 142.0, 112.9 (d, *J* = 191.5 Hz), 101.7, 95.4 (d, *J* = 190.3 Hz), 81.4 (d, *J* = 5.0 Hz), 81.3 (d, *J* = 6.3 Hz), 71.2 (d, *J* = 16.4 Hz), 61.9 (d, *J* = 27.7 Hz), 38.2, 37.7 (d, *J* = 26.5 Hz), 27.9, 26.5, 16.1 (d, *J* = 6.3 Hz). ^19^F NMR (470 MHz, DMSO-*d*_6_) δ –186.39 to –186.21 (m). ^31^P NMR (202 MHz, DMSO-*d*_6_) δ 20.21. HRMS calc. for C_24_H_35_FN_2_O_10_PSi [M+H]^+^ 561.2008, found 561.2017.

#### Synthesis of compound 6

To a solution of **5** (1.2 g, 2.14 mmol) in CH_2_Cl_2_ (10 mL) and 5-(*S*-ethylthio)-1*H*-tetrazole (0.25 M in CH_3_CN; 10.3 ml, 2.57 mmol) was added dropwise 2-cyanoethyl *N,N,N′,N′*-tetraisopropylphosphorodiamidite (0.816 ml, 2.57 mmol) at 0°C. The mixture was stirred at rt for 3 h. The reaction mixture was quenched with saturated NaHCO_3_ (aq.) and washed with saturated NaHCO_3_ (aq.), water and brine, dried (Na_2_SO_4_), and concentrated under vacuum. The crude residue was purified by column chromatography on silica gel (70–100% ethyl acetate) to give compound **6** as a white foam (1.14 g, 70%). ^1^H NMR (500 MHz, CD_3_CN) δ 9.28 (brs, 1H), 7.17–7.20 (m, 1H), 6.63–6.73 (m, 1H), 6.06 (dd, *J* = 17.6 and 19.63 Hz, 0.55H), 5.87–5.95 (m, 0.45H), 5.56–5.63 (m, 5H), 4.70–5.01 (m, 3H), 3.58–3.86 (m, 4H), 2.69 (t, *J* = 6.1 Hz, 1.1H), 2.62–2.65 (m, 0.9H), 1.83–1.86 (m, 1H), 1.50–1.55 (m, 1H), 1.31–1.34 (m, 1H), 1.15–1.21 (m, 30H). ^13^C NMR (101 MHz, CD_3_CN) δ 177.7, 177.7, 177.7, 177.6, 164.20, 155.0, 154.9, 151.8, 143.5, 143.3, 119.7, 119.6, 116.1, 116.1, 116.0, 114.2, 114.1, 114.1, 114.1, 103.0, 103.0, 97.2, 96.5, 96.5, 95.3, 94.6, 94.6, 82.8, 82.7, 82.7, 82.7, 82.6, 82.6, 75.2, 75.1, 75.1, 75.1, 74.9, 74.8, 64.8, 64.5, 64.2, 64.0, 59.8, 59.6, 59.4, 59.2, 44.4, 44.3, 44.3, 44.2, 39.5, 39.1, 39.0, 38.8, 38.8, 38.7, 38.6, 38.4, 38.3, 29.3, 27.3, 25.2, 25.1, 25.1, 25.0, 24.9, 21.1, 21.0, 21.0, 17.4, 17.3, 17.3, 17.2. ^19^F NMR (470 MHz, CD_3_CN) δ –184.91 to –184.65 (m). ^31^P NMR (202 MHz, CD_3_CN) δ 151.90 (d, *J* = 10.9 Hz), 151.03 (d, *J* = 9.2 Hz), 19.64, 19.56. HRMS calc. for C_33_H_52_FN_4_O_11_P_2_ [M+H]^+^ 761.3092, found 761.3101.

#### Synthesis of triphosphates 13 and 14

The triphosphates were prepared following previously described methods (56,57). Compounds **9** and **10** were synthesized on an ABI-394 synthesizer in 1-μmol scale on universal support. A solution of 0.25 M 5-(*S*-ethylthio)-1*H*-tetrazole in acetonitrile (CH_3_CN) was used as the activator. The solutions of the synthesized 2′-F-NMC amidites (**7** and **8**) (37) were 0.15 M in anhydrous CH_3_CN. The oxidizing reagent was 0.02 M I_2_ in THF/pyridine/H_2_O. The detritylation reagent was 3% dichloroacetic acid in CH_2_Cl_2_. Compounds **11** and **12**, which have 5′ triphosphate groups, were synthesized on an ABI-394 synthesizer. A solution of 1 M diphenyl phosphite in pyridine was used for phosphitylation of 5′-hydroxyl group. A solution of 0.1 M triethylammonium bicarbonate (TEAB) in H_2_O/CH_3_CN was used for hydrolysis to obtain the *H*-phosphonate intermediates. The obtained *H*-phosphonates were treated with a solution of 1 M imidazole/1 M *N,O*-bis(trimethylsilyl)acetamide in CBrCl_3_/CH_3_CN/Et_3_N. Reaction with a solution of 0.25 M tributylammonium pyrophosphate in DMF/CH_3_CN resulted in 5′-triphosphate compounds **11** and **12**. After completion of the automated synthesis, the triphosphates were manually released from support and deprotected using a mixture of 28% aqueous ammonia/EtOH [3:1 (v/v)] for 5 h at 55°C. After filtration through a nylon syringe filter (0.45 μm), the crude triphosphates were purified using anion-exchange high-performance liquid chromatography (IEX-HPLC) using an appropriate gradient of mobile phase (H_2_O and 0.5 M TEAB in H_2_O). The collected triphosphates were then lyophilized and treated with DOWEX 50DX2 (Na^+^ form). The obtained triphosphates **13** and **14** were quantified by measuring the absorbance at 260 nm using the following extinction coefficients: T/U, 7.92 M^−1^cm^−1^; C, 6.57 M^−1^ cm^−1^.

#### Compound 13

^1^H NMR (400 MHz, D_2_O) δ 7.97 (d, *J* = 8.0 Hz, 1H), 5.96 (d, *J* = 8.0 Hz, 1H), 4.96 (d, *J* = 17.5 Hz, 1H), 4.66–4.84 (m, 2H), 4.54 (dd, *J* = 5.2 and 11.2 Hz, 1H), 3.80 (dd, *J* = 5.0 and 11.2 Hz, 1H), 1.74–1.77 (m, 1H), 1.25–1.28 (m, 1H), 1.00–1.06 (m, 1H). ^31^P NMR (162 MHz, D_2_O) δ -10.1 (d, *J* = 19.4 Hz), –10.6 (d, *J* = 19.4 Hz), –22.4 (d, *J* = 19.4 and 19.4 Hz). MS; *m*/*z* 415 [M–H]^−^.

#### Compound 14

^1^H NMR (400 MHz, D_2_O) δ 8.05 (d, *J* = 7.6 Hz, 1H), 6.18 (d, *J* = 7.6 Hz, 1H), 4.98 (d, *J* = 17.2 Hz, 1H), 4.54–4.75 (m, 3H), 3.77 (dd, *J* = 5.0 and 11.2 Hz, 1H), 1.74–1.77 (m, 1H), 1.27–1.29 (m, 1H), 0.98–1.04 (m, 1H). ^31^P NMR (162 MHz, D_2_O) δ –10.07 (d, *J* = 19.4 Hz), –10.63 (d, *J* = 19.4 Hz), –22.48 (t, *J* = 19.4 Hz). MS; *m*/*z* 414 [M–H]^−^.

### Oligonucleotide synthesis

Oligonucleotides were synthesized on an ABI-394 and MerMade 192 synthesizer at 1-μmol scale using universal or custom supports. A solution of 0.25 M 5-(*S*-ethylthio)-1*H*-tetrazole in acetonitrile (CH_3_CN) was used as the activator. The phosphoramidite solutions of commercially available standard RNA/DNA amidites, and synthesized 2′-F-NMC amidites were 0.15 M in anhydrous CH_3_CN or 9:1 CH_3_CN:DMF (2′-OMe-C, 2′-OMe-U). The oxidizing reagent was 0.02 M I_2_ in THF/pyridine/H_2_O. The detritylation reagent was 3% dichloroacetic acid in CH_2_Cl_2_. Standard protocols were used for cleavage and deprotection after synthesis of oligonucleotides on the ABI-394. Crude oligonucleotides were purified using strong anion exchange and phosphate buffers (pH 8.5) containing sodium bromide.

After the trityl-off synthesis using the MerMade 192, columns were incubated with 150 μl of 40% aqueous MeNH_2_ for 30 min at room temperature, and the solution was drained via vacuum into a 96-well plate. After repeating the incubation and draining with a fresh portion of aqueous MeNH_2_, the plate containing the crude oligonucleotides was sealed and shaken at room temperature for an additional 60 min to completely remove all protecting groups. In the case of RNA, the 2′-hydroxyl was deprotected by treating with Et_3_N·3HF at 60°C for 60 min. Precipitation of the crude oligonucleotides was accomplished via the addition of 1.2 ml of CH_3_CN:EtOH (9:1, v/v) to each well, followed by centrifuged at 3000 rpm for 45 min at 4°C. The supernatant removed from each well, and the pellets were resuspended in 950 μl of 20 mM aqueous NaOAc. Each crude solution was desalted over a GE Hi-Trap desalting column (Sephadex G25 Superfine) using water to elute the final oligonucleotide products.

Oligonucleotide concentrations were calculated based on absorbance at 260 nm and the following extinction coefficients: A, 13.86 M^−1^cm^−1^; T/U, 7.92 M^−1^cm^−1^; C, 6.57 M^−1^cm^−1^ and G, 10.53 M^−1^cm^−1^. The purity and identity of oligonucleotides were verified by analytical anion exchange chromatography and mass spectrometry (Supplementary Table S1 and Supplementary Figure S1–S3), respectively.

### *In vitro* screening

#### Cell culture and transfection

Primary mouse hepatocytes were obtained from Life Technologies and cultured in Williams E Medium with 10% fetal bovine serum. Transfection was carried out in 384-well plates by adding 4.9 μl of Opti-MEM plus 0.1 μl of Lipofectamine RNAiMax (Invitrogen) per well to 5 μl of each siRNA duplex at the desired concentration. After incubation at room temperature for 20 min, 40 μl of complete growth media containing 5000 cells was added to the siRNA mixture. Cells were incubated for 24 h prior to RNA isolation. A similar procedure was followed for the transfection of 10,000,000 cells, scaled accordingly. Dose response experiments were done using eight 6-fold serial dilutions over the range of 75 pM to 20 nM or 187.5 pM to 50 nM.

#### RNA isolation

RNA was isolated using a Dynabeads mRNA Isolation Kit (Invitrogen). Cells were lysed in 75 μl of Lysis/Binding Buffer containing 3 μl of beads per well and mixed for 10 min on an electrostatic shaker. Buffers were prepared according to the manufacturer's protocol. The washing steps were automated on a Biotek EL406 using a magnetic plate support. Beads were washed (90 μl) once in buffer A, once in buffer B, and twice in buffer E, with aspiration steps between washes.

#### cDNA synthesis

cDNA synthesis was accomplished with the High-capacity cDNA Reverse Transcription Kit (Applied Biosystems). A mixture of 1 μl of 10× buffer, 0.4 μl of 25× dNTPs, 1 μl of random primers, 0.5 μl of reverse transcriptase, 0.5 μl of RNase inhibitor and 6.6 μl of water per reaction were added per well. Plates were sealed, agitated for 10 min on an electrostatic shaker, and then incubated at 37°C for 2 h. Following this, the plates were agitated at 80°C for 8 min.

#### Real-time PCR

cDNA (2 μl) was added to a master mix containing 0.5 μl mouse GAPDH TaqMan Probe (Applied Biosystems, Cat.# 4308313), 0.5 μl of mouse ApoB or PTEN TaqMan probes (Applied Biosystems, Cat.# Mm01545156_m1 and Mm01212532_m1, respectively) and 5 μl of Lightcycler 480 probe master mix (Roche) per well in a 384-well plate (Roche). Real-time PCR was done in an ABI 7900HT RT-PCR system (Applied Biosystems) using the ΔΔCt (RQ) assay. Each duplex and concentration were tested in four biological replicates. To calculate relative fold change, real-time data were analyzed using the ΔΔCt method and normalized to assays performed with cells transfected with 10 nM nonspecific siRNA. IC_50_ values were calculated using a four-parameter fit model using XLFit.

### Evaluation of silencing in mice

All studies were conducted using protocols consistent with local, state, and federal regulations, as applicable, and approved by the Institutional Animal Care and Use Committee at Alnylam Pharmaceuticals. Mice received a single subscapular subcutaneous injection of 1 mg/kg siRNA, prepared in an injection volume of 10 μl per g body weight in PBS. At the indicated time pre- or post-dosing, animals were anesthetized with isofluorane and blood was obtained via retroorbital bleed. TTR protein was quantified by ELISA from serum isolated from whole blood. The ELISA was performed according to the manufacturer's protocol (ALPCO, 41-PALMS-E01) after a 3025-fold dilution of the serum samples. Data were normalized to prebleed TTR levels. All samples were assayed in duplicate, and each data point is the average of all the mice within each cohort (*n* = 3). Data were analyzed using a two-way ANOVA with a Tukey posthoc test for multiple comparison in GraphPad Prism.

### Luciferase assay

COS-7 cells were cultured at 37°C, 5% CO_2_ in Dulbecco's modified Eagle's medium supplemented with 10% fetal bovine serum. Cells were co-transfected in 96-well plates (15 000 cells/well) with 10 ng luciferase reporter plasmid and 0.64 pM to 50 nM siRNA in 5-fold dilutions using 2 μg/ml Lipofectamine 2000 (Thermo Fisher Scientific) according to the manufacturer's instructions. Cells were harvested 48 h after transfection for the dual luciferase assay (Promega) according to the manufacturer's instructions. The on-target and off-target reporters were generated by Blue Heron Biotech by cloning into the psiCHECK2 vector between XhoI and NotI restriction sites in the 3′-UTR of *Renilla luciferase*. The on-target reporter plasmid contained a single site perfectly complementary to the guide strand in the 3′-UTR of *Renilla luciferase* (5′-AAAACAGTGTTCTTGCTCTATAA-3′). The off-target reporter plasmid contained four tandem seed-complementary sites (5′-GCTCTATAA-3′) separated by a 19-nucleotide spacer (5′-TAATATTACATAAATAAAA-3′) in the 3′-UTR of *Renilla luciferase* (58–62). Both the on-target and off-target regions were flanked at the 5′ end by 5′-ATAAACAAGGTTTGACATCAATCTAGCTATATCTTTAAGAATGATAAACT-3′ and at the 3′ end by 5′-GACATTGGTGAGGAAAAATCCTTTGGCCGTTTCCAAGATCTGACAGTGCA-3′. Both plasmids co-expressed firefly luciferase as a transfection control.

### Molecular modeling

Coordinates of the miR-20a:Ago2 complex (PDB ID code 4f3t) (63) the complex of Ago2 bound to guide and target RNAs (PDB ID code: 4w5t) (47) and the DNA hairpin with NMC T modifications (C4′-*exo* pucker; PDB ID code: 4dkz) (44) were retrieved from the Protein Data Bank (www.rcsb.org). The 2′-F-NMC U residue with an C2′-*exo* pucker was taken from the coordinates of a 2′-F-NMC:RNA hybrid duplex model built earlier (37). All model building was performed with the program UCSF Chimera (64) using the match option for initial superimpositions and adapting backbone torsion angles in order to generate optimal overlays between 5′- and 3′-phosphate groups of RNA and 2′-F-NMC nucleotides. All models were optimized with the program Amber (14ff) (65) as implemented in UCSF Chimera by using both steepest descent and conjugate gradient minimization procedures until conversion.

### Polymerase incorporation assay

Human mitochondrial RNA polymerase (POLRMT) was purchased from Indigo Biosciences (Cat# MV100-40). Fluorophore-labeled RNA primer (5′-(Atto 425)UUUUGCCGCGCC-3′) was synthesized in house; DNA templates were obtained from IDT (template sequences were 5′-GGGAATCAT**G**GGCGCGGC-3′ and 5′-GGGAATTGC**A**GGCGCGGC-3′ for primer extension with cytosine- and uracil-containing species). Reaction conditions for the POLRMT incorporation assays were as follows: DNA template (200 nM), 12-mer 5′-(Atto-425)-labeled RNA primer (25 nM), POLRMT (300 nM), substrate (1 mM or 100 μM NTP or 2′-F-NMC NTP **13** and **14**), reaction buffer (20 mM Tris–HCl, pH 7.5, 10 mM MgCl_2_, 10 mM DTT, 0.05% Tween-20), 37°C for 30 min. Reactions were quenched by the addition of 25 mM EDTA, followed by denaturation at 90°C for 5 min. The reaction mixtures were diluted with water to 1 nM primer and analyzed by IEX-HPLC (λ_excitation_: 436 nm, λ_emission_: 485 nm) using a DNAPac200 4 × 250 mm column. Buffer A: 20 mM sodium phosphate, 10% ACN, pH 11; buffer B: 20 mM sodium phosphate, 10% ACN, 1 M NaBr, pH 11. The flow was 1 ml/min and the gradient was 15–35% buffer B over 2 min, then 35–40% buffer B over 16 min, then 40–60% buffer B over 15 min.

Purified human mitochondrial DNA polymerase subunit gamma (POLG), exonuclease activity-deficient, was obtained from the laboratory of Prof. William Copleand at the National Institute of Environmental Health Science, NIEHS, NC, USA. Fluorophore-labeled DNA primer (5′-(Atto 425)d[CGATATTCACAAAG]-3′) was synthesized in house; DNA templates were obtained from IDT (template sequences were 5′-d[CATGCTCTAACCGCGCTTTGTGAATATCG]-3′ and 5’-d[CATGCTCTAACCCGACTTTGTGAATATCG]-3’ for primer extension with cytosine- and uracil-containing species). Reaction conditions for the POLG incorporation assays were as follows: DNA template (100 nM), 14-mer 5′-fluorophore (Atto-425) labeled DNA primer (100 nM), POLG exo-mutant (40 units), substrate (1 mM or 100 μM NTP or 2′-F-NMC NTP **13** and **14**), reaction buffer (20 mM Tris–HCl, pH 8.0, 10 mM MgCl_2_, 2 mM β-mercaptoethanol, 0.1 mg/ml bovine serum albumin), 37°C for 30 min. Reactions were quenched by the addition of 25 mM EDTA, followed by denaturation at 80°C for 5 min. The reaction mixtures were diluted with water to 1 nM primer and analyzed by IEX-HPLC (λ_excitation_: 436 nm, λ_emission_: 485 nm) using a DNAPac200 4 × 250 mm column. Buffer A: 20 mM sodium phosphate, 10% ACN, pH 11; buffer B: 20 mM sodium phosphate, 10% ACN, 1 M NaBr, pH 11. The flow was 1 ml/min and the gradient was 15–35% buffer B over 2 min, then 35–40% buffer B over 16 min, then 40–60% buffer B over 15 min.

## RESULTS AND DISCUSSION

### Synthesis of 5′-(*E*)-VP 2′-F-NMC uridine phosphoramidite

**Scheme 1.**
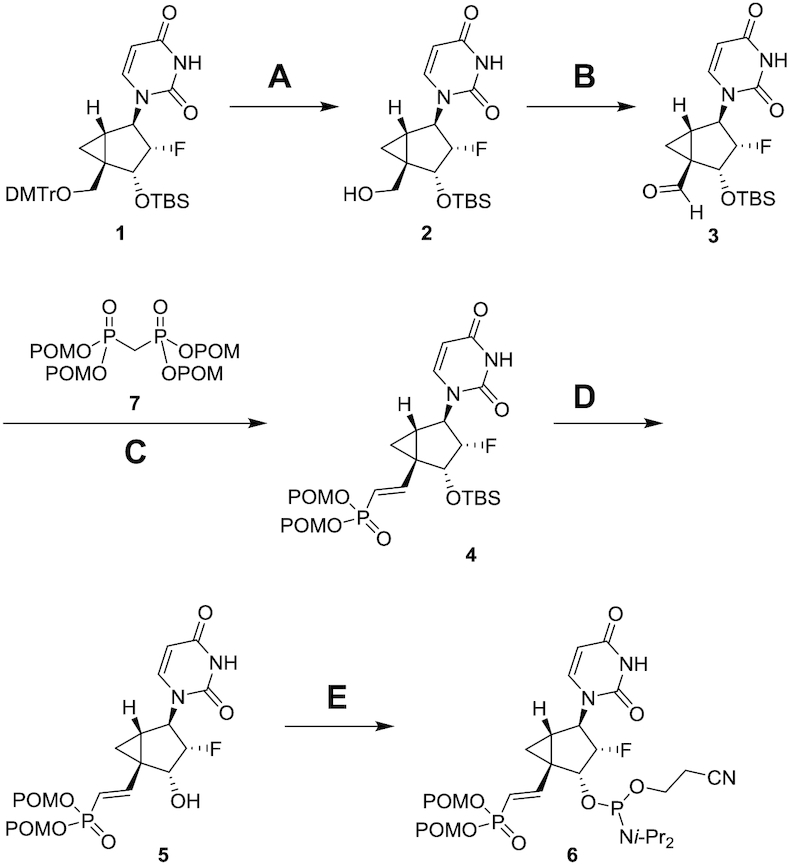
Synthesis of 5′-(*E*)-VP 2′-F-NMC uridine phosphoramidite **6** (**A**) 80% AcOH aq., 67%; (**B**) Dess-Martin periodinane, pyridine, CH_2_Cl_2_, 99%; (**C**) NaH, THF, 93%; (**D**) HCO_2_H/H_2_O (1:1), 93%; (**E**) 2-cyanoethyl *N,N,N*′*,N*′-tetraisopropylphosphorodiamidite, 5-(*S*-ethylthio)-1*H*-tetrazole, CH_2_Cl_2_, 70%. DMTr = 4,4′-dimethoxytrityl, TBS = *tert*-butyldimethylsilyl, POM = pivaloyloxymethyl, *i*-Pr = isopropyl.

The 5′-(*E*)-VP-modified 2′-F-NMC uridine phosphoramidite was synthesized as depicted in Scheme 1 starting with the fully protected 2′-F-NMC uridine nucleoside **1**, which was synthesized as we previously reported (37). The 5′-DMTr protection was removed by treatment with 80% acetic acid to obtain the 3′-*O*-TBS-protected uridine derivative **2**. Oxidation of the 5′-OH group of **2** with Dess-Martin periodinane afforded the aldehyde **3**. Subsequent Horner-Wadsworth-Emmons olefination of the aldehyde **3** with tetrakis[(pivaloyloxy)methyl] methylenediphosphonate **7** with a procedure similar to that previously described (55) led to the 5′-(*E*)-bis(pivaloyloxymethyl)-phosphonate **4** in 93% yield as a single isomer. The presence of the cyclopropane ring may sterically hinder generation of the (*Z*) isomer on the transition state, leading to the observed selectivity. The 3′-TBS protection of bisPOM phosphonate **4** was removed under formic acid and water (1:1) conditions to yield the alcohol **5**. Phosphitylation of **5** gave the desired phosphoramidite **6**, which was used for oligonucleotide synthesis under standard solid-phase synthesis conditions.

### Synthesis of pyrimidine 2′-F-NMC NTPs

**Scheme 2.**
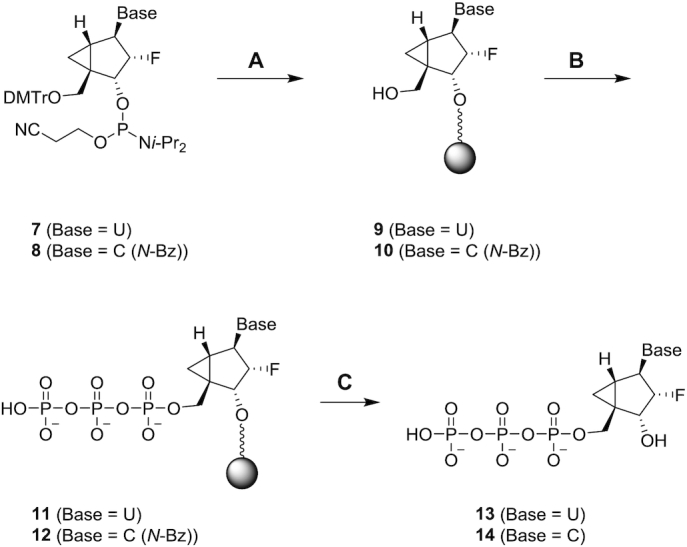
Synthesis of 2′-F-NMC uridine and cytidine triphosphates **13** and **14**. Steps (**A**) and (**B**) were performed on an ABI-394 oligonucleotide synthesizer essentially as described (54,55); (**C**) NH_4_OH/EtOH, 55°C, 5 h. DMTr = 4,4′-dimethoxytrityl, *i*-Pr = isopropyl, Bz = benzoyl.

The syntheses of 2′-F-NMC uridine triphosphate (**13**; UTP) and cytidine triphosphate (**14**; CTP) started from 2′-F-NMC uridine and cytidine phosphoramidites (**7** and **8**) and were synthesized as we previously reported (37) as depicted in Scheme 2. The phosphoramidites **7** and **8** were linked to CPG support using an ABI-394 synthesizer, and DMTr groups were removed to obtain CPG-supported nucleotides **9** and **10**, respectively. Compounds **9** and **10** were treated with four successive reactions on an ABI-394 synthesizer to give 5′-triphosphates **11** and **12**: The first reaction was 5′-phosphitylation of the 5′-hydroxyl groups using diphenyl phosphite; the second was hydrolysis of the resulting phenyl 5′-*H*-phosphonate diesters to the corresponding 5′-*H*-phosphonate monoesters using aqueous triethylammonium bicarbonate buffer; the third was silylation and oxidation of the 5′-*H*-phosphonates to give activated 5′-phosphorimidazolidates using a mixture of *N*,*O-*bis(trimethylsilyl)acetamide, bromotrichloromethane, and imidazole; and the fourth was nucleophilic substitution of the imidazole from the 5′-phosphorimidazolidates by the pyrophosphate anion using the tributylammonium salt of pyrophosphate (56,57). The resulting compounds **11** and **12** were cleaved from the CPG support and benzoyl group of the cytidine derivative **12** was removed by treating with a mixture of ammonium hydroxide and EtOH to produce the 2′-F-NMC UTP **13** and CTP **14**, which were used for polymerase incorporation assay.

### *In vitro* gene silencing mediated by siRNAs containing 2′-F-NMC

We previously reported that 2′-F-NMC causes almost no thermal destabilization of an RNA:RNA duplex (37). Further, the 2′-F-NMC imparted more 3′-exonuclease stability than did the 2′-F-RNA modification when substituted at the 3′ end of an oligonucleotide and more 5′-exonuclease stability when substituted at the penultimate position from the 5′ end (37). Based on these encouraging biophysical properties, the impact of 2′-F-NMC modification on gene silencing was assessed within the context of a previously described siRNA duplex targeting mouse *Ttr* mRNA (Figure 1) (13,18). The parent siRNA, which is fully modified with 2′-OMe and 2′-F and has strategically placed phosphorothioate linkages, was designed to provide a balance between stability toward nucleolytic degradation and productive association with Ago2 (13,18,66). As unmodified RNA duplexes are metabolically very unstable and therapeutically useless, the fully modified parents strand enables a clinically relevant comparison between siRNAs with and without 2′-F-NMC.

**Figure 3.**
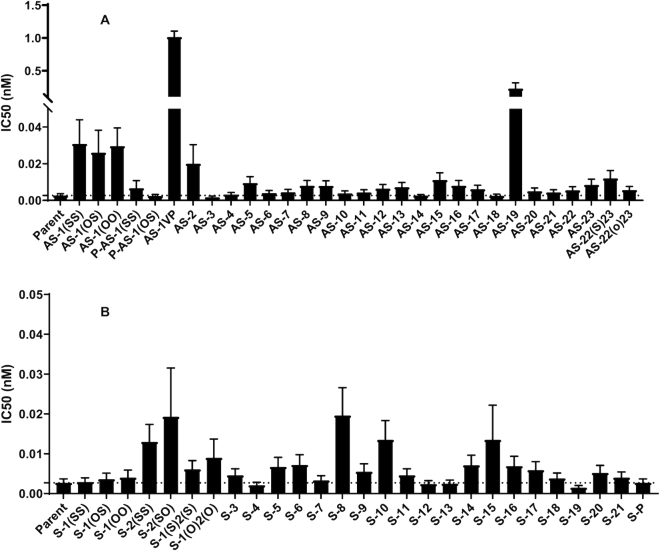
IC_50_ values (nM) of siRNAs targeting *Ttr* with single 2′-F-NMC substitutions relative to the parent siRNA in the (**A**) guide strand and (**B**) passenger strand. Activity was determined after transient transfection into primary mouse hepatocytes. See Supplementary Table S1 and S2 (Supplementary Information) for detailed information. In siRNA designations, OS, OO and SS refer to the first two linkages with O indicative of phosphate and S of phosphorothioate; the P refers to the presence of a 5′-phosphate.

To determine the positional effect of 2′-F-NMC incorporation on silencing activity, we evaluated siRNAs modified at single positions with 2′-F-NMC throughout both the guide and passenger strands replacing either the 2′-OMe or 2′-F (Supplementary Table S1). To evaluate the potential of this set of modified duplexes to silence *Ttr* gene expression *in vitro*, siRNAs were transfected into primary mouse hepatocytes, and IC_50_ values were determined. The incorporation of 2′-F-NMC was generally well tolerated in both the guide (Figure 3A) and passenger (Figure 3B) strands with some exceptions including several positions previously shown to be intolerant to chemical modifications (17,67–69). These positions, 1 and 2 on the guide strand and 10 on the passenger strand, likely must be native nucleotides or 2′-F-RNA due to their importance in Ago2 association of the guide strand (positions 1 and 2) and passenger strand cleavage and removal (position 10) (47,63,70).

In the guide strand, substitution of position 1 with 2′-F-NMC decreased potency relative to the parent siRNA. The siRNA with a 2′-F-NMC at position 1, but a 5′ phosphate at the 5′ end (with either a phosphorothioate or a phosphodiester linkage) had an IC_50_ values similar to the parent. This indicates that the 2′-F-NMC is a poor substrate for 5′-phosphorylating enzymes and synthetic placement of a phosphate or phosphorothioate is needed for the activity. Interestingly, the siRNA with a 2′-F-NMC at position 1 modified with the metabolically stable phosphate mimic 5′-(*E*)-VP was considerably less potent than the siRNA with 2′-F-NMC at position 1 and a 5′-monophosphate.

Of particular interest are positions 6 and 7 of the guide strand, where it has been shown that the interaction with an isoleucine side chain of Ago2 causes a kink in the guide RNA structure (47,63). The siRNAs with 2′-F-NMC at either of these positions were active; thus, this modification is tolerated at the kink. Substitutions with 2′-F-NMC at positions 10, 11 and 12 were also well tolerated, suggesting that the activity of the central region of guide strand is not governed by the Ago2 interactions, but rather by the hybridization of the guide strand with target mRNA. 2′-F-NMC has an A-type sugar conformation and binding affinity similar to the parent. Excessive high-affinity binding to the target mRNA might interfere with the product release and catalytic silencing function. In contrast to positions 1 and 2, there are no reliable structural models that provide a clear picture of the interactions between the PIWI domain of Ago2 and positions 9–12 of the guide strand (17). Surprisingly, various chemistries, such as amide-backbone-containing siRNAs (71), *S*-glycol nucleic acids (*S*-GNAs) (72) and altritol nucleic acids (ANAs) (73) are tolerated at these positions. We believe that as long as the nucleobase is able to bind to target mRNA the slicer can exert its silencing function.

The siRNA with two adjacent 2′-F-NMC residues at positions 22 and 23 in guide strand (with either a phosphorothioate or a phosphodiester linkage) was marginally less potent than the parent. The sugar ring of the terminal guide strand residue packs against the backbone of residues H336, T337 and Y338 from the PAZ domain of Ago2 (63). The 2′-OH of the ribose forms a hydrogen bond to the keto oxygen of H336 and the 3′-OH forms hydrogen bonds to keto oxygen and amino group of Y338. Fluorine at the 2′-position of precludes this hydrogen bond formation. Moreover, the 4′-oxygen of the 3′-terminal ribose is in van der Waals contact with the side chain of T337. The cyclopropane ring (C7′) of 2′-F-NMC results in a more closely spaced sugar moiety vis-à-vis the threonine side chain and will require slight reorientations of either sugar or side chain or both. The side chain has limited degrees of freedom as it is part of a hydrophobic cluster that also includes the side chains of V267, Y279, V281, L328, V330 and L339. Both the loss of a hydrogen bond as well as the steric constraints might account for the reduced potency of a guide with 2′-F-NMC at position 23. The side chain of R277 is engaged in a hydrogen bond to the hydroxyl group of Y279 and can reach the 4′-oxygen of the ribose of residue 22 (4 Å distance in the Ago2 complex with miR-20a (63)). This electrostatically favorable configuration is prevented by the presence of the cyclopropane ring in 2′-F-NMC, providing a qualitative rationalization of the slight loss in potency observed for a guide with 2′-F-NMC at position 22.

In the passenger strand, loss of potency was observed when a single 2′-F-NMC was incorporated at position 2, 8 or 10. Surprisingly, 2′-F-NMC was tolerated at positions 11 and 12 of the passenger strand; we had expected that close proximity to position 10, which is important during the cleavage reaction of the passenger strand and loading (47,63,70), might mean that substitutions at these positions would considerably reduce activity. siRNAs with 2′-F-NMC at positions 1, 4 and 19 of the passenger strand were also almost as potent as the parent siRNA. The placement of two 2′-F-NMC residues at positions 1 and 2 in the passenger strand (with either a PO or a PS linkage) resulted in slight loss of gene silencing activity compared to that of parent. There is no information from structures on Ago2 interactions with the 5′ end of the passenger strand. In the crystal structure of the complex between the Ago1 PAZ domain and a short duplex with a 3′-terminal overhang, nucleotides S1 and S2 are close to residues Q348 and R349 (which are located in the same positions in Ago2 PAZ (74). The keto group of Q348 is positioned underneath the ribose of S1 and the amino group participates in a hydrogen bond to the phosphate of position S2.

### *In vivo* gene silencing mediated by 2′-F-NMC-modified guide strand containing siRNAs

**Figure 4.**
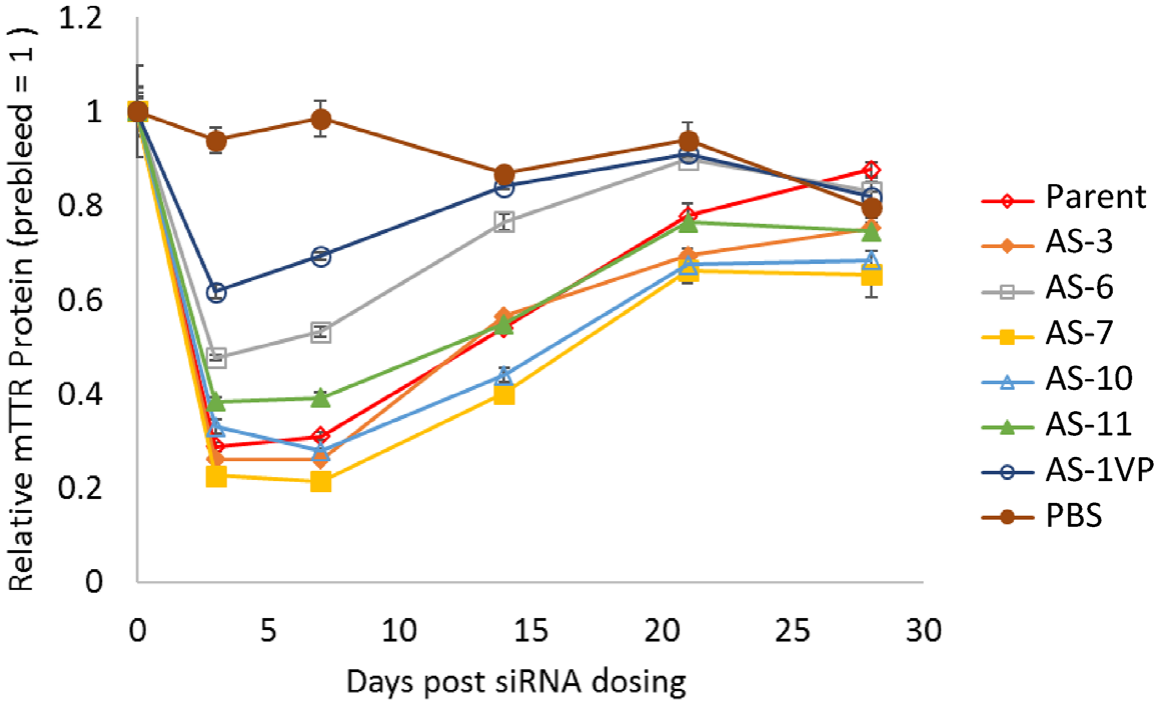
Relative amount of circulating TTR (compared to pre-dose levels) as a function of days after treatment with parent or 2′-F-NMC-modified siRNA. Mice were treated with PBS, parent siRNA, or 2′-F-NMC-modified siRNA duplexes at 1 mg/kg. TTR protein levels were quantified in serum. See Supplementary Table S1 (Supplementary Information) for sequence information.

Several of the siRNA duplexes with 2′-F-NMC residues in the seed region of guide strand and the siRNA with a 5′-(*E*)-VP and a 2′-F-NMC at position 1 of the guide strand were evaluated for efficacy in mice. Mice were treated subcutaneously with siRNA at a dose of 1 mg/kg (the approximate ED_80_ of the parent siRNA (18)), and circulating TTR protein levels were monitored over a period of 28 days (Figure 4). Administration of the parent duplex led to a 70% reduction of TTR levels on day 7 compared to the PBS-treated controls. As expected based on *in vitro* results, modification of position 1 with 5′-(*E*)-VP 2′-F-NMC led to only 30% reduction of TTR levels on day 7. siRNAs with 2′-F-NMC at position 3, 10 or 11 had similar or slightly better activity than the parent siRNA. In contrast, modification at position 6 of the guide strand reduced TTR levels only by about 50% on day 7. On the other hand, the siRNA with 2′-F-NMC at position 7 in the seed region of the guide strand reduced TTR levels by 80% on day 7 and was the top performer among those evaluated.

### On-target and off-target activities of siRNAs modified with 2′-F-NMC

**Figure 5.**
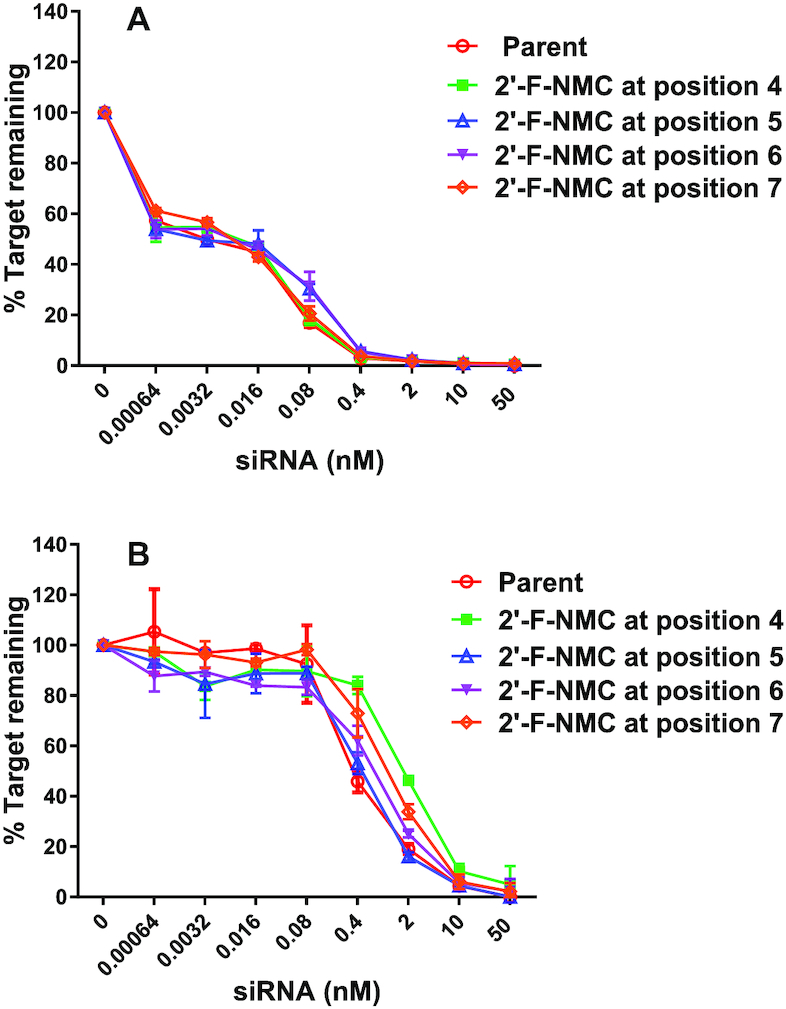
(**A**) On-target and (**B**) off-target silencing activities of parent siRNA and siRNAs with 2′-F-NMC-modified guide strands in a luciferase reporter assay. Luciferase reporter plasmids were co-transfected with indicated siRNAs into COS-7 cells. The cells were harvested at 48 h after transfection for the dual luciferase reporter assay. In the dual luciferase reporter assay, the activities of firefly and *Renilla* luciferases are measured sequentially from a single sample. The % target remaining was calculated by dividing the ratio of *Renilla* to firefly luciferase signal at each siRNA concentration by the ratio in the absence of siRNA.

Seed-mediated off-target activity has been reported to contribute to hepatoxicity of siRNAs in rats (75). To reduce such off-target effects, thermally destabilizing modifications such as *S*-GNA have been incorporated into the seed region of siRNA duplexes (72,75). *S*-GNA can efficiently mitigate seed-mediated, off-target effects without reducing on-target potency. To test the impact of 2′-F-NMC in the seed region on on- and off-target activities of the siRNA guide strand, we utilized reporter plasmids containing either a single fully complementary site or four tandem seed-complementary sites in the 3′-UTR of *Renilla luciferase* (58–62). These reporter constructs co-expressed firefly luciferase with no siRNA binding sites as normalization control. Following transfection into COS-7 cells, the on- and off-target activities were assessed by determining *Renilla* to firefly luciferase ratio in the presence of increasing concentrations of the siRNA, as measured by the dual luciferase assay. Consistent with primary hepatocyte transfection data (Figure 3A), we confirmed that 2′-F-NMC does not impact siRNA on-target activity with a luciferase construct containing a single 3′-UTR binding site complementary to the guide strand of the *Ttr*-targeted siRNA (Figure 5A). The off-target activities of the parent duplex and the modified duplexes were similar (Figure 5B). Thus, 2′-F-NMC neither improves off-target activity nor does it make it worse. This correlates well with the hybridization properties of 2′-F-NMC which are similar to 2′-F and 2′-OMe.

### Modeling analysis of interactions of a 2′-F-NMC-containing guide strand with Ago2

**Figure 6.**
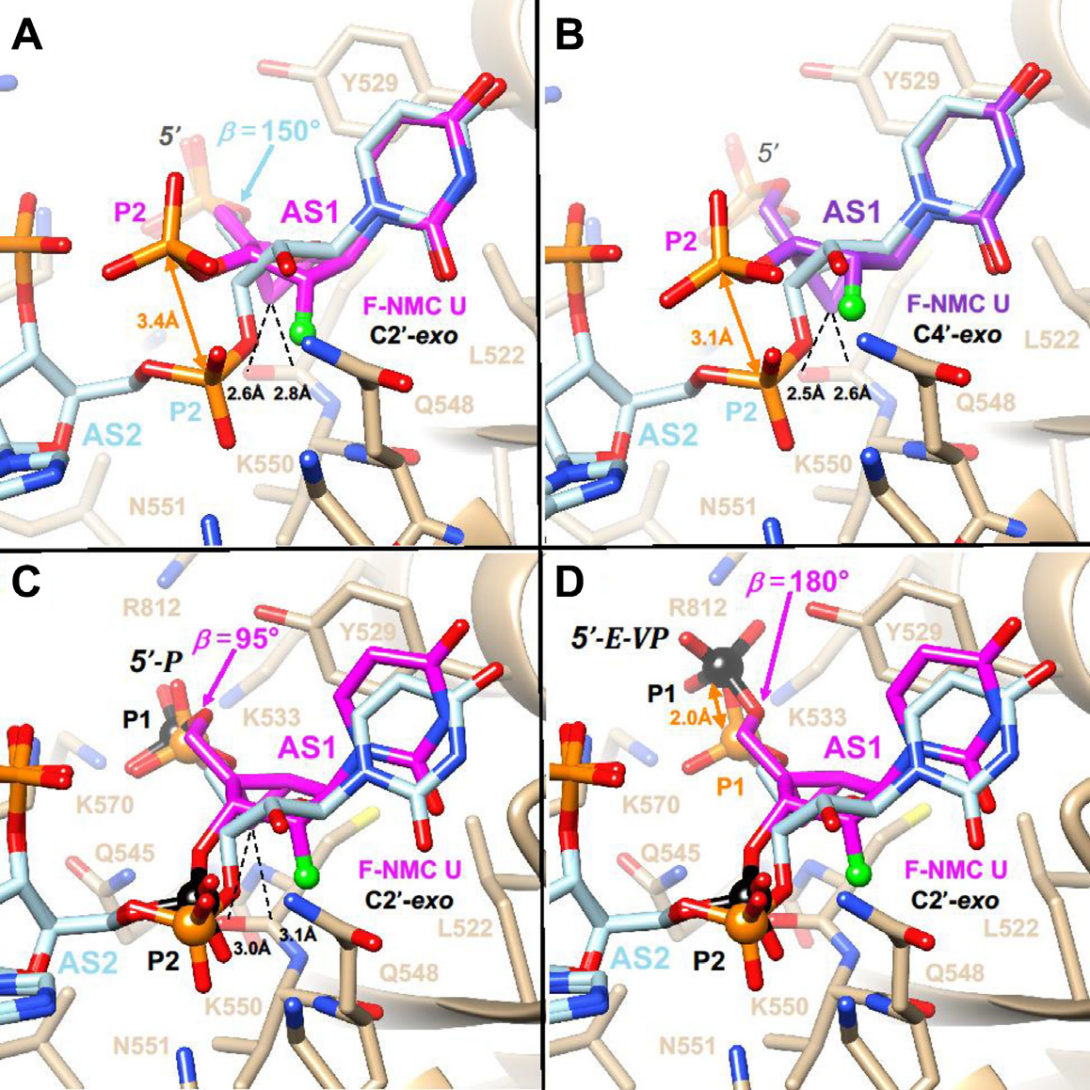
Models of the interactions with Ago2 with 2′-F-NMC at position 1 of guide strand of the siRNA. (**A**) 2′-F-NMC with a C2′-*exo* pucker at position 1. (**B**) 2′-F-NMC with a C4′-*exo* pucker at position 1. (**C**) Energy-minimized conformation and orientation of 5′-phosphate-2′-F-NMC at position 1. (**D**) Energy-minimized conformation and orientation of 5′-(*E*)-VP-2′-F-NMC at position 1. Carbon atoms of native nucleotides are colored in light blue. Those of 2′-F-NMC in the C2′-*exo* conformation and of 2′-F-NMC in the C4′-*exo* conformation are colored in magenta and purple, respectively. F2′ is shown as a light green sphere. Ago2 is drawn as a beige ribbon with selected side chains displayed and labeled. The deviations between the positions of the 3′ phosphates of 2′-F-NMC and the corresponding phosphate in RNA (panels A and B) and those of 5′ phosphate and 5′-(*E*)-VP (panel D) are highlighted with orange arrows. Selected contacts between the 2′-F-NMC cyclopropane ring (C7′) and Ago2 main chain atoms are indicated with dashed lines, and 5′ and 3′ phosphates of 2′-F-NMC in panels C and D are highlighted as black spheres.

To gain insight into the altered activities displayed by siRNAs with 2′-F-NMC modifications at positions 1, 2, 6 or 7 of the guide strand relative to the parent siRNA, we modeled the interactions between Ago2 and 2′-F-NMC-containing oligonucleotides. To do so we primarily relied on the crystal structure of miR-20a bound to Ago2 (63). For the model with the modified nucleotide at position 1 of the guide strand, we used 2′-F-NMC uridine in either the C2′-*exo North* conformation as observed in our NMR study and in a model duplex (37) or the C4′-*exo northeast* conformation as observed for NMC thymidine in the crystal structure of a DNA hairpin (44). 2′-F-NMC uridines with these puckers were individually superimposed on the uridine at position 1 of miR-20a using the coordinates of the crystal structure of this miRNA bound to Ago2 (PDB ID code 4f3t) (63). The ribose of the nucleotide at position 1 of the guide strand in the complex with Ago2 adopts an atypical south C2′-*endo* or C1′-*exo* pucker (17). Thus, 2′-F-NMC U in the preferred *north* or *northeast* conformations can in principle bind such that 5′-phosphate and nucleobase orientations are maintained relative to the native guide strand, but the 3′-phosphate position deviates significantly from that seen in the parent complex (Figure 6A, B). The scenario with the 5′ phosphate bound properly necessitates that the backbone undergo a sharp turn between the first and the second nucleotide. Alternatively, the 5′ phosphate could be altered relative to the conformation of the unmodified strand with base and 3′-phosphate orientations more or less preserved (not shown). The misaligned first bridging phosphate would be unable to interact with the Ago2 side chains Q548 and N551 (Figure 6A, B). Thus, either arrangement would interfere with the optimal interaction between the 5′ phosphate and the MID domain.

We next adjusted the β (O5′-C5′), ϵ (C3′-O3′) and ζ (O3′-P) torsion angles of the 2′-F-NMC residue at position 1 and tilted the nucleotide slightly to overlay the 5′ and 3′ phosphates and the uracil base with those of the native uridine residue. This orientation was then energy-minimized with Amber 14ff in the UCSF Chimera Suite (64) until convergence. In the optimized conformation and orientation, the fit with the ribonucleotide is quite good, and stacking with Y529 and hydrogen-bonding interactions between the 3′ phosphate and Q548 and N551 are maintained (Figure 6C). This is consistent with the observation that 2′-F-NMC in combination with a 5′ phosphate at position 1 had potency similar to the parent siRNA. However, in order for the 5′ phosphate to sit properly between K533, Q545, K570 and R812 requires the β torsion angle in 2′-F-NMC to contract to 95° (Figure 6C). When the phosphate was replaced with a 5′-(*E*)-VP moiety, which required adjustment of the C5′-O5′ bond length slightly and opening the β angle to 180°, the phosphate shifted by 2 Å, and the optimal orientation relative to the basic side chains of Ago2 was lost (Figure 6D). This explains the observed loss of potency relative to the parent siRNA when position 1 of the guide strand was 2′-F-NMC in combination with a 5′-(*E*)-VP moiety.

**Figure 7.**
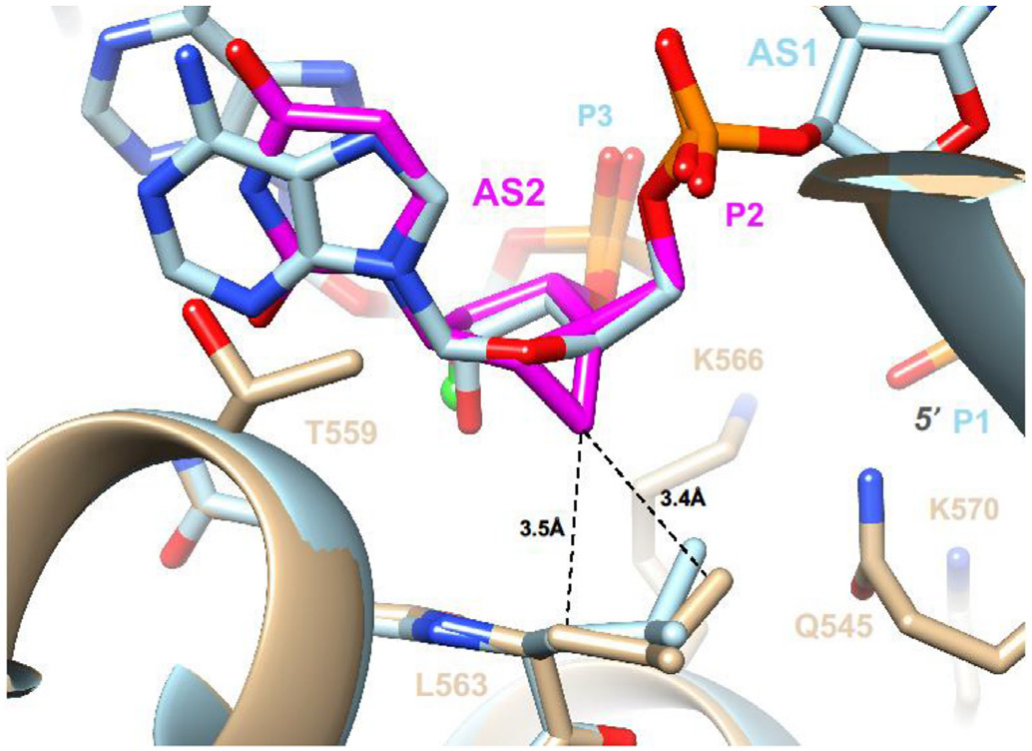
Interaction between Ago2 and 2′-F-NMC with a C2′-*exo* pucker at position 2 of the guide strand. The color codes match those in Figure 6. Energy minimization of the complex with the 2′-F-NMC-modified RNA resulted in slight adjustments of the L563 side chain (tan, model; light blue, crystal structure of native complex). However, neither side chain nor sugar can move farther apart to relieve slightly short contacts between cyclopropane ring (C7′) and the L563 methylene and methyl group.

At position 2 of the guide strand, the 2′-F-NMC U residue with a *north* pucker can be neatly superimposed on adenosine located at that site in the crystal structure of the complex, with matching positions between 5′- and 3′-phosphates of the native and modified strands (Figure 7). However, as for insertion of a 2′-F-NMC nucleotide at AS1 (Figure 6A, B), the cyclopropane ring lies rather close to an Ago2 residue, in this case the side chain of L563 that protrudes from an α-helical portion of the MID domain. Thus, the origin of the intolerance to replacing a ribonucleotide by a 2′-F-NMC residue at AS2 is similar to loss of activity seen for 2′-*O*-methyl modification. The only modifications known to be tolerated at position 2 of the guide strand other than the natural 2′-OH are 2′-deoxy and 2′-F (17).

**Figure 8.**
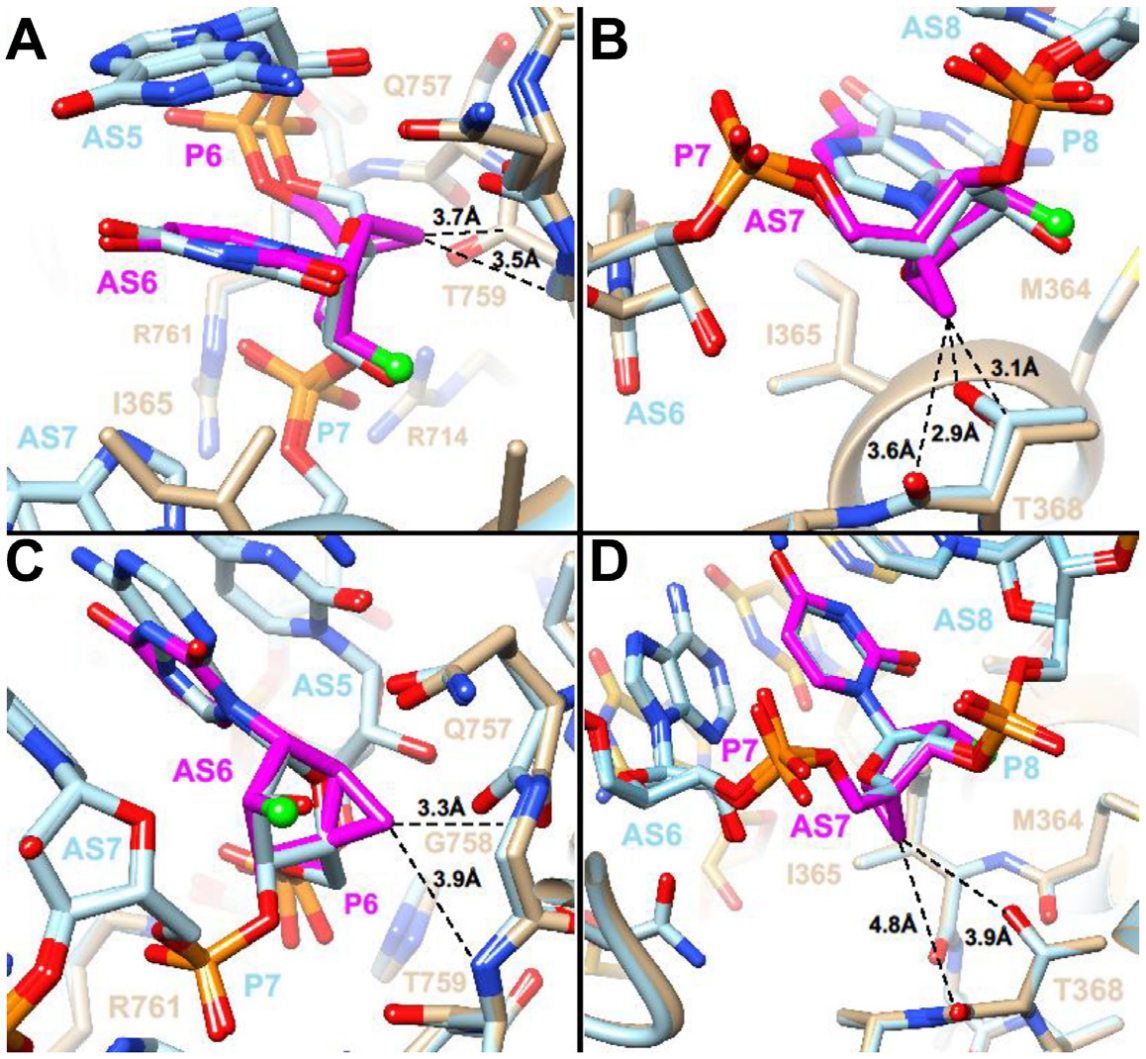
Models of the interactions with Ago2 of 2′-F-NMC at positions 6 and 7 of guide siRNA based on complexes with miR-20a (PDB ID code: 4f3t) and a guide-passenger strand duplex (PDB ID code: 4w5t). (**A**) Model obtained upon substitution with 2′-F-NMC at position 6 of miR-20a. (**B**) Model obtained upon substitution with 2′-F-NMC at position 7 of miR-20a. (**C**) Model obtained upon substitution with 2′-F-NMC at position 6 of guide strand. (**D**) Model obtained upon substitution with 2′-F-NMC at position 7 of guide strand. All models were energy-minimized. The color codes match those in Figure 6. Carbon atoms of RNA strands and protein main and side chains in the refined models and crystal structures are colored in tan and light blue, respectively. Carbon atoms of passenger strand nucleotides visible in panels C and D are yellow.

In crystal structures of Ago2 bound to miR-20a (63) and of Ago2 bound to a seed duplex composed of guide and passenger strand (47), the guide strand has a kink between nucleotides 6 and 7. This kink is particularly pronounced in the complex with miRNA, where the side chain of I365 is inserted between the nucleobases of guide strand residues at positions 6 and 7 (Figure 8A, B). The *in vitro* potencies of guide strands with either position 6 or 7 replaced by 2′-F-NMC are similar to that of the parent strand, indicating that the analog is well tolerated at those sites.

In summary, models of complexes with 2′-F-NMC inserted into the siRNA guide strand either at position 6 or 7 show that the modified nucleotide can be accommodated at both sites. Analyses of the complexes with miR-20a (Figure 8A, B) and with a guide-passenger duplex (Figure 8C, D) show that the interactions between guide and Ago2 differ somewhat. Thus, 2′-F-NMC at position 6 adopts slightly different orientations relative to G758 and T759 (Figure 8A, C). Similarly, 2′-F-NMC at position 7 adopts slightly different orientations relative to T368 (Figure 8B, D). However, except for a slightly short contact between C7′ and the keto group of T368 (Figure 8B), 2′-F-NMC does not interfere with the kink and adjustments of guide siRNA relative to Ago2 in that region. At present, the interaction between the PAZ domain and the terminal modification of the guide strand is not fully understood. It is possible that increased lipophilicity, size and increased conformational rigidity may impair the interaction with the Ago2 PAZ domain and thus account for the activity loss observed for 2′-F-NMC substitution at position 19.

### Analysis of use of 2′-F-NMC NTPs as substrates for POLRMT and POLG

**Figure 9.**
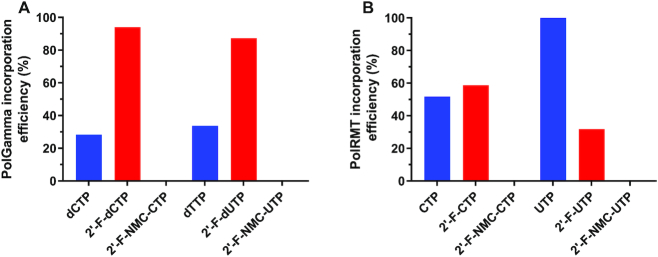
(**A**) Incorporation of 2′-deoxy, 2′-F, and 2′-F-NMC monomers into the primer in the POLG primer extension assay. (**B**) Incorporation of ribo, 2′-F and 2′-F-NMC NTP monomers into the primer in the POLRMT primer extension assay. Incorporation efficiency was calculated as 100% minus the % area of the remaining primer based on the integration of the fluorescence signal at λ_ex_ = 436 nm and λ_em_ = 485 nm. Average value is the result of two replicate experiments. See Supplementary Figure S4 and S5 and Supplementary Table S3 and S4 (Supplementary Information) for experimental details. Note that the values for 2′-F-dNTP were obtained from previously published data (20).

Incorporation of 2′-F-NMC, RNA, DNA, and 2′-F NTPs were evaluated in a previously reported *in vitro* primer extension assays catalyzed by the human mitochondrial RNA polymerase POLRMT and human mitochondrial DNA polymerase POLG (20). Incorporation of 2′-F-NMC U and C NTPs (Scheme 2) were tested. Briefly, a 5′-fluorescently labeled primer, template, and a single NTP were incubated with POLRMT for 30 minutes or with POLG for 35 min, and the primer extension reaction was monitored via fluorescence detection IEX-HPLC. As expected, native NTPs (rCTP, rUTP, dCTP and dTTP) were efficiently incorporated by the polymerases (Figure 9). The 2′-F-dNTPs were also used as substrates, albeit with lower efficiency than canonical NTPs except in the case of cytosine nucleosides as previously described (20). Interestingly, however, the 2′-F-NMC analogues **13** and **14** were not substrates for POLRMT or POLG, whereas 2′-F monomers were more efficiently incorporated than canonical ribo- and deoxy-NTPs by POLRMT (Figure 9). These data suggest that due to the very poor substrate character of the 2′-F-NMC NTPs, any potential toxicities due to recognition by mitochondrial polymerases will not be observed with 2′-F-NMC.

## CONCLUSIONS

In summary, we systematically evaluated potencies of siRNA duplexes with single 2′-F-NMC modifications in a therapeutically relevant gene target both *in vitro* and *in vivo*. Our *in vitro* analysis of gene silencing demonstrates that 2′-F-NMC is accommodated without loss in efficacy at most positions of both the guide and the passenger strands. Incorporation of 2′-F-NMC into the guide strand at position 1 resulted in a loss of the *in vitro* activity that was recovered when the siRNA was modified with both a 5′ phosphate and the 2′-F-NMC. This confirmed that 2′-F-NMC is a poor substrate for natural kinases and suggested that this modification will not have mitochondrial toxicity, which is associated with nucleotide analogs that are recognized by kinases (20). In further support of this hypothesis, in *in vitro* assays with POLRMT and POLG, 2′-F-NMC NTPs were not substrates for the polymerases.

The phosphate mimic 5′-(*E*)-VP on the 2′-F-NMC at the position 1 was detrimental to efficacy in mice. Guide strands carrying 5′-(*E*)-VP on simple RNA mimics, (e.g. 2′-OMe) or even larger RNA mimics like 2′-*O*-NMA, that can adopt a C2′-*endo* conformation when imposed by the enzyme, were effectively loaded into the RISC due to formation of hydrogen bonds with the MID domain of Ago2 (17,45–47,54). Conformationally restricted nucleotides bearing bicyclic scaffolds, such as 2′-F-NMC, may impair these hydrogen-bonding interactions due to a conflict between the stereoelectronic demands of 5′-(*E*)-VP with the MID domain of Ago2 and the steric demands of the bicyclic 2′-F-NMC, which cannot adopt a C2′-*endo* conformation. Although in most cases results were similar *in vitro* and *in vivo*, there was a small difference in activity when the 2′-F-NMC modification was located at position 6 of the guide strand. At present we are not able to account for this variation. The siRNA with 2′-F-NMC at position 7 in the seed region outperformed all siRNAs tested including the parent siRNA. Remarkably, substitutions with 2′-F-NMC at guide positions 10, 11, and 12 were also well accepted.

In the off-target evaluation using the luciferase reporter assay, 2′-F-NMC residues at the positions 4, 5, 6, or 7 in the seed region of the guide strand had minimal impact on miRNA-like seed-based off-target activity. We recently reported that thermodynamically destabilizing modifications like *S*-GNA can reduce off-target gene-silencing effects when placed in the seed region of the passenger strand, as the modification destabilizes binding to off-target mRNA (72). Unlike *S*-GNA, 2′-F-NMC is not thermodynamically destabilizing (37) and therefore had off-target effects similar to the parent siRNA. Thus, the 2′-F-NMC modification is similar to the recently reported binding affinity-neutral ANA modification with regards to off-target gene silencing (73).

Our results indicate that 2′-F-NMC holds promise as a modification for siRNA due to increased lipophilicity and improved nuclease resistance compared to the commonly used 2′-F siRNA modification (37) and to lack of recognition by kinases and polymerases. Specifically, 2′-F-NMC is not a substrate for mitochondrial RNA and DNA polymerases POLRMT and POLG, indicating that metabolites should not be toxic (20,76). Evaluation of pharmacokinetic and preclinical safety studies in rodents and pharmacology and duration studies in higher species are needed. Moreover, 2′-F-NMC deserves further evaluation in other modes of nucleic acids-based therapeutics beyond RNAi that employ other bicyclic (LNA, BNAs and cEt) and tricyclic nucleic acid modifications (77–80).

## Supplementary Material

gkab050_Supplemental_File

## Notes

Present address: Derek O'Flaherty, Department of Chemistry, University of Guelph, Guelph, Ontario, N1G 2W1, Canada.
