# Supplementary material for: siRNAs containing 2′-fluorinated Northern-methanocarbacyclic (2′-F-NMC) nucleotides: in vitro and in vivo RNAi activity and inability of mitochondrial polymerases to incorporate 2′-F-NMC NTPs
Source: Nucleic Acids Res. 2021 Feb 12;49(5):2435–49. doi: 10.1093/nar/gkab050 (PMC7969009; doi:10.1093/nar/gkab050)
Supplement: gkab050_Supplemental_File [file gkab050_supplemental_file.pdf]

Supporting Information

siRNAs Containing 2′-Fluorinated *Northern*-Methanocarbacyclic (2′-F-NMC) Nucleotides: *In Vitro* and *In Vivo* RNAi Activity and Inability of Mitochondrial Polymerases to Incorporate 2′-F-NMC NTPs

Masaaki Akabane-Nakata[1,*], Namrata D. Erande[1], Pawan Kumar[1], Rohan Degaonkar[1], Jason Gilbert[1], June Qin[1], Martha Mendez[1], Lauren Blair Woods[1], Yongfeng Jiang[1], Maja M. Janas[1], Derek K. O'Flaherty[1], Ivan Zlatev[1], Mark Schlegel[1], Shigeo Matsuda[1], Martin Egli[2], and Muthiah Manoharan[1,*]

[1] Alnylam Pharmaceuticals, 675 West Kendall Street, Cambridge, Massachusetts, 02142, United States
[2] Department of Biochemistry, School of Medicine, Vanderbilt University, Nashville, Tennessee 37232, United States

* To whom correspondence should be addressed. Tel: 617-551-8319; Email: mmanoharan@alnylam.com; correspondence can also be addressed to mnakata@alnylam.com

Table of Contents

^{1}H, ^{13}C, ^{19}F, and ^{31}P NMR spectra for the new compounds S3

Mass components of 2′-F-NMC NTP S25

Oligonucleotide characterization S27

Table S1. Sequences and mass spectroscopy characterization of siRNA strands S27

Figure S1. LCMS spectrum and mass components of oligonucleotide AS-1VP S30

Figure S2. Example of LCMS Spectrum of oligonucleotide S-16 .... S31

Figure S3. Example of LC-MS spectrum of siRNA duplex S-16 .... S31

In vitro screening S32

Table S2. *In vitro* gene silencing by duplexes modified with single 2′-F-NMC nucleotide substitution targeting mTTR mRNA S32

Polymerase incorporation assay S36

Figure S4. Incorporation of canonical ribonucleotides and 2′-F-NMC nucleotides in the POLRMT primer extension assay S36

Table S3. Incorporation of 2′-F-NMC monomers in the POLRMT primer extension assay . S37

Figure S5. Incorporation of canonical 2′-deoxyribonucleotides and 2′-F-NMC nucleotides in the PolGamma primer extension assay S38

Table S4. Incorporation of 2′-F-NMC monomers in the PolGamma primer extension assay S39

References S40

^{1}H, ^{13}C, ^{19}F and ^{31}P NMR spectra for the new compounds

^{1}H NMR spectrum of compound **2** in DMSO-d_6

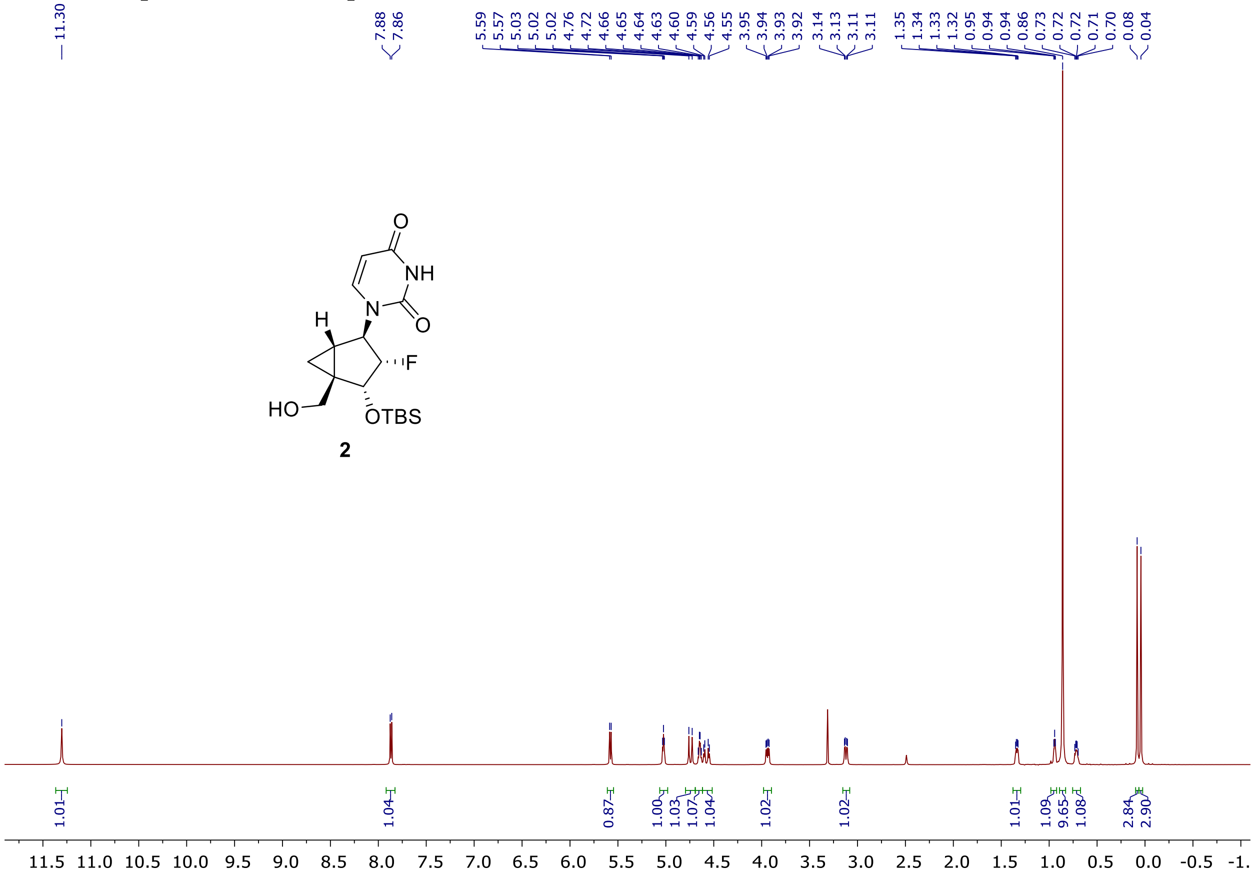

^{13}C NMR spectrum of compound **2** in DMSO-d_6

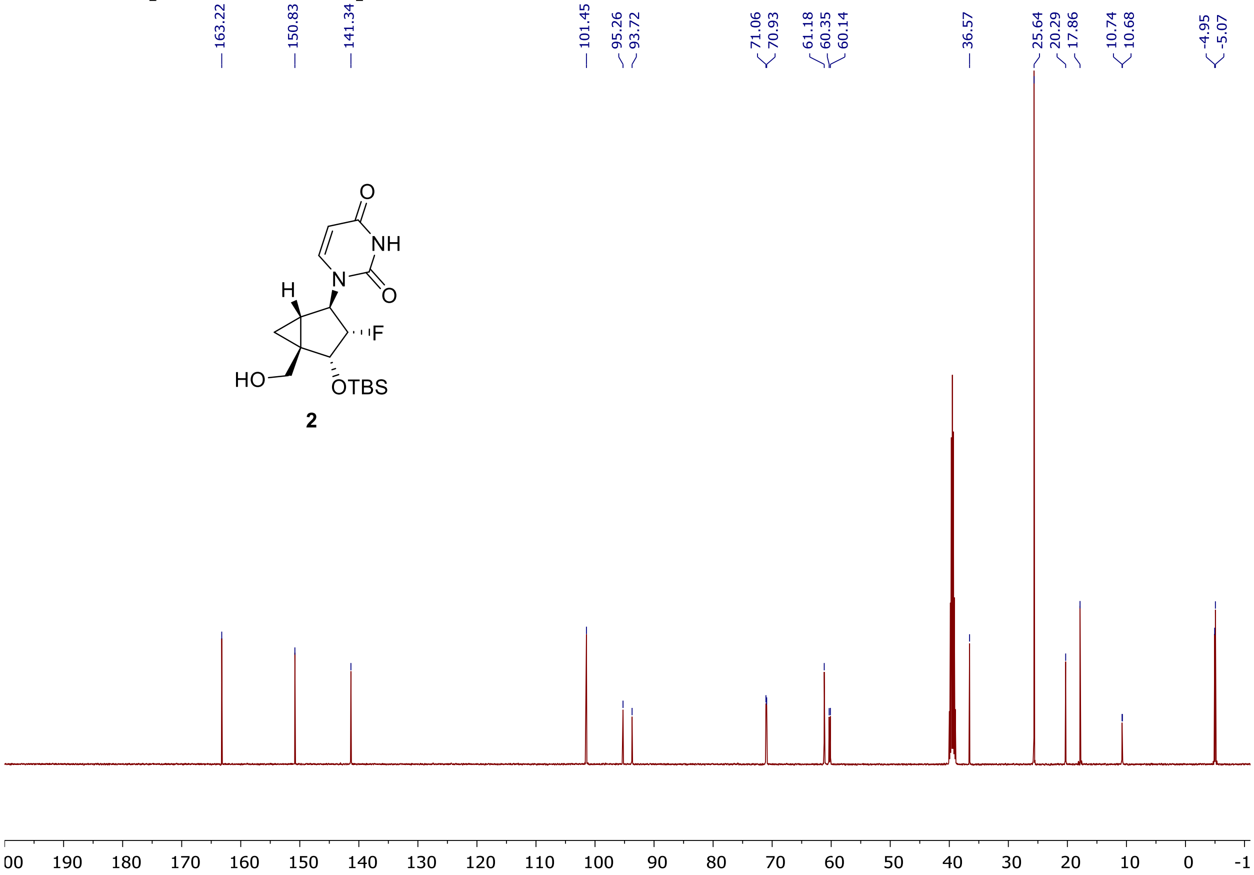

^{19}F NMR spectrum of compound **2** in DMSO-d_6

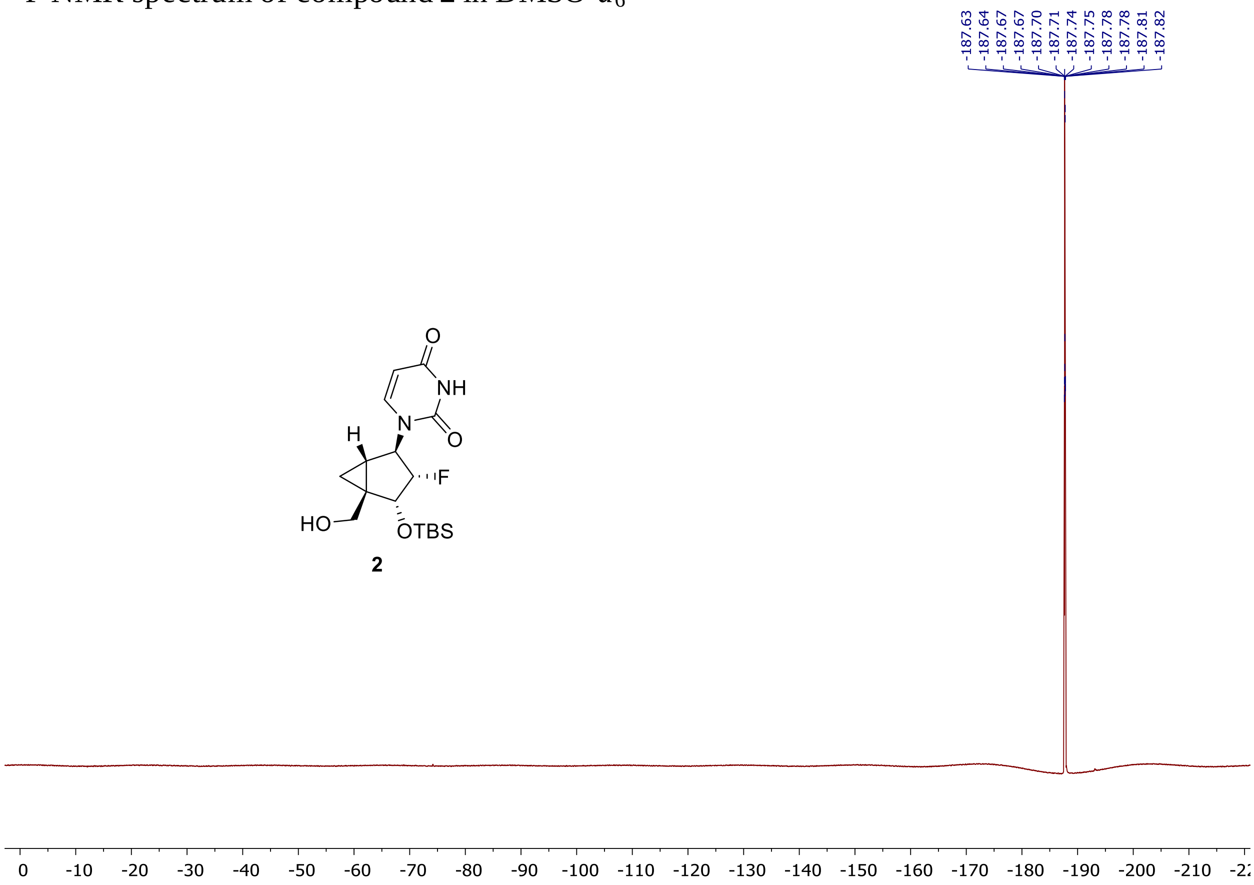

^{1}H NMR spectrum of compound **3** in DMSO-d_6

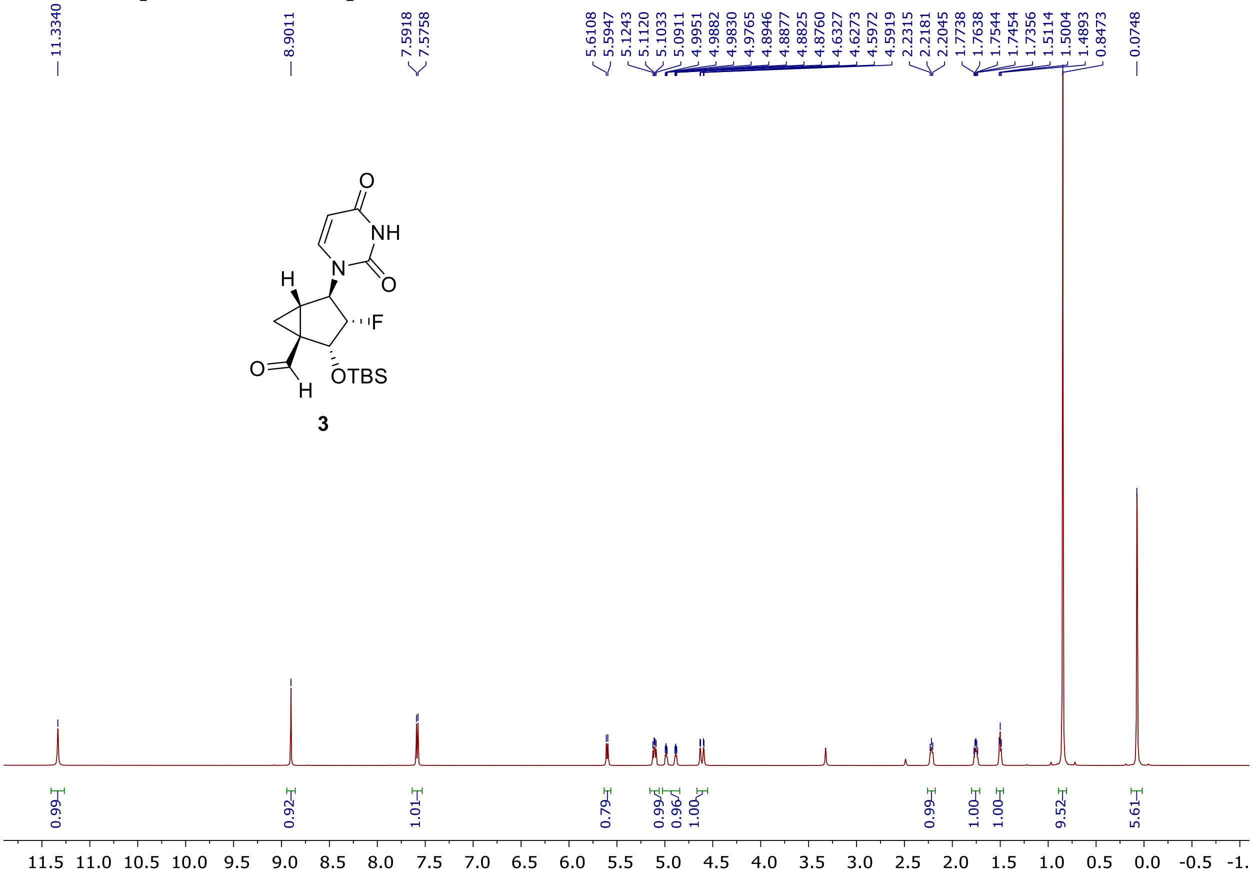

^{13}C NMR spectrum of compound **3** in DMSO-d_6

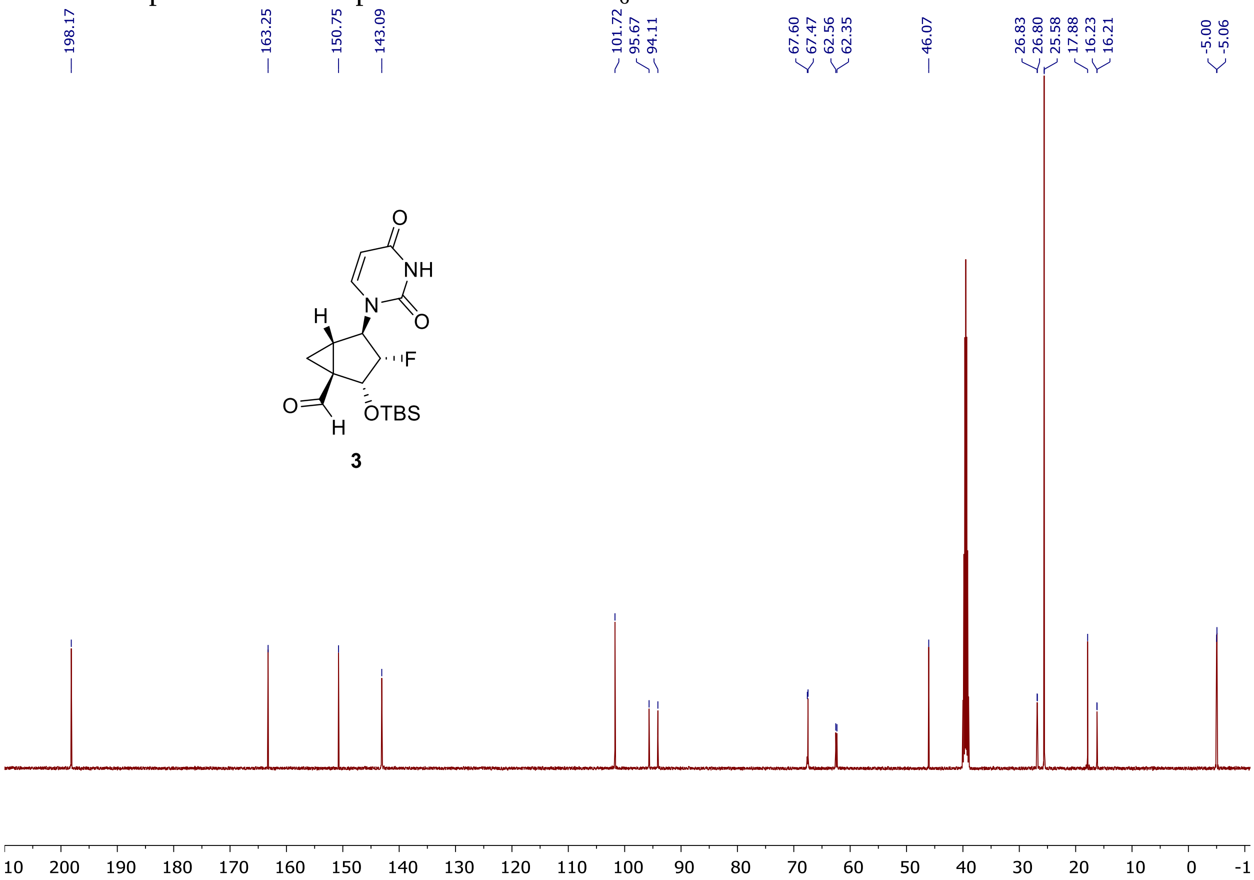

^{19}F NMR spectrum of compound **3** in DMSO-d_6

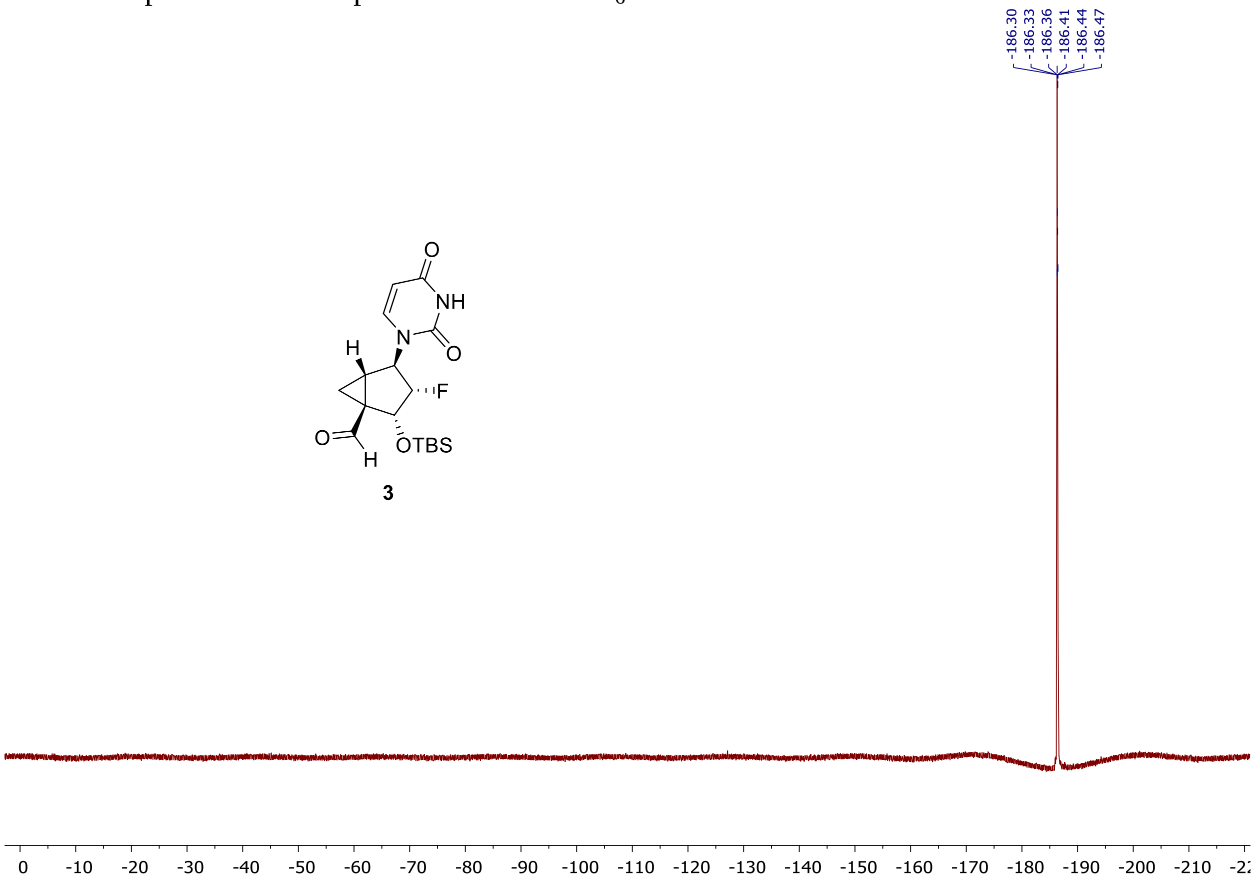

^{1}H NMR spectrum of compound **4** in DMSO-d_6

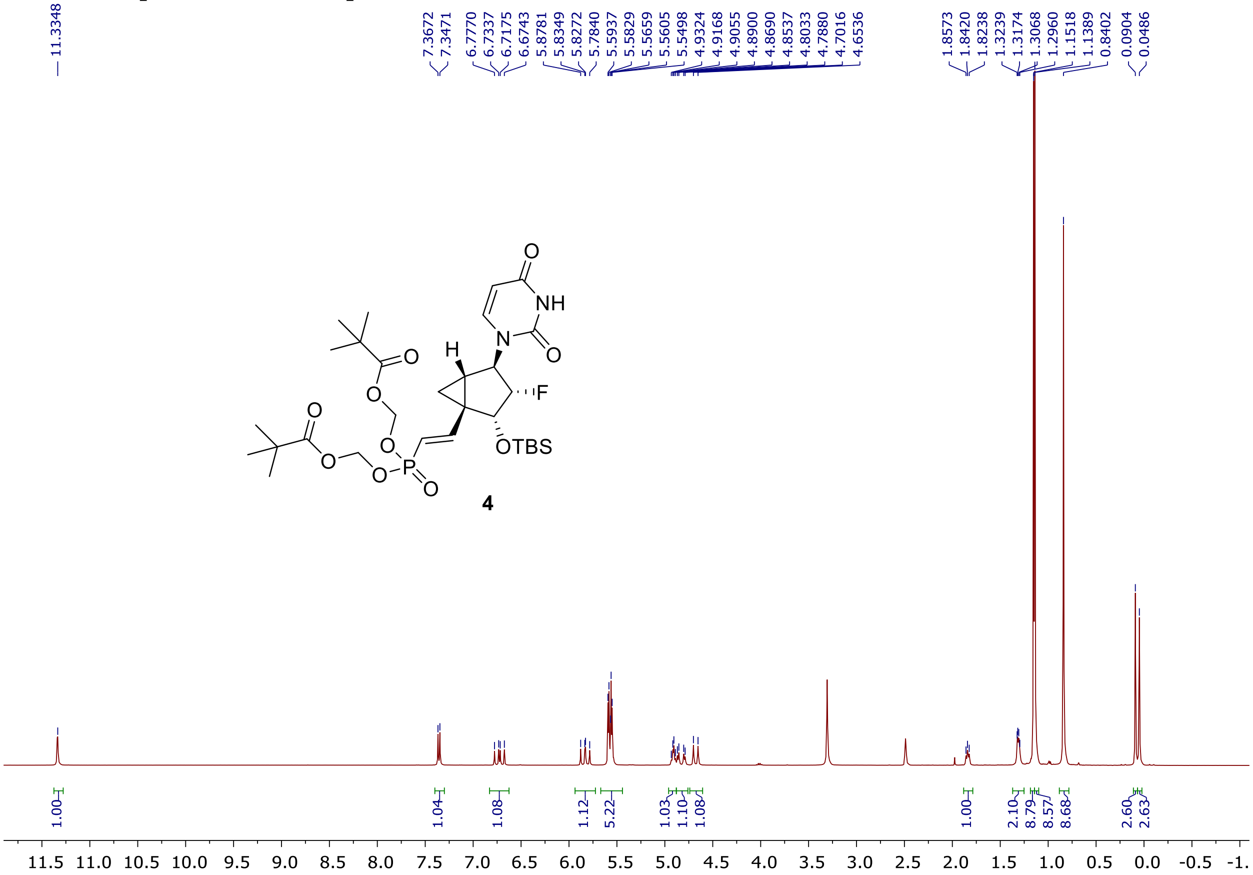

^{13}C NMR spectrum of compound **4** in DMSO-d_6

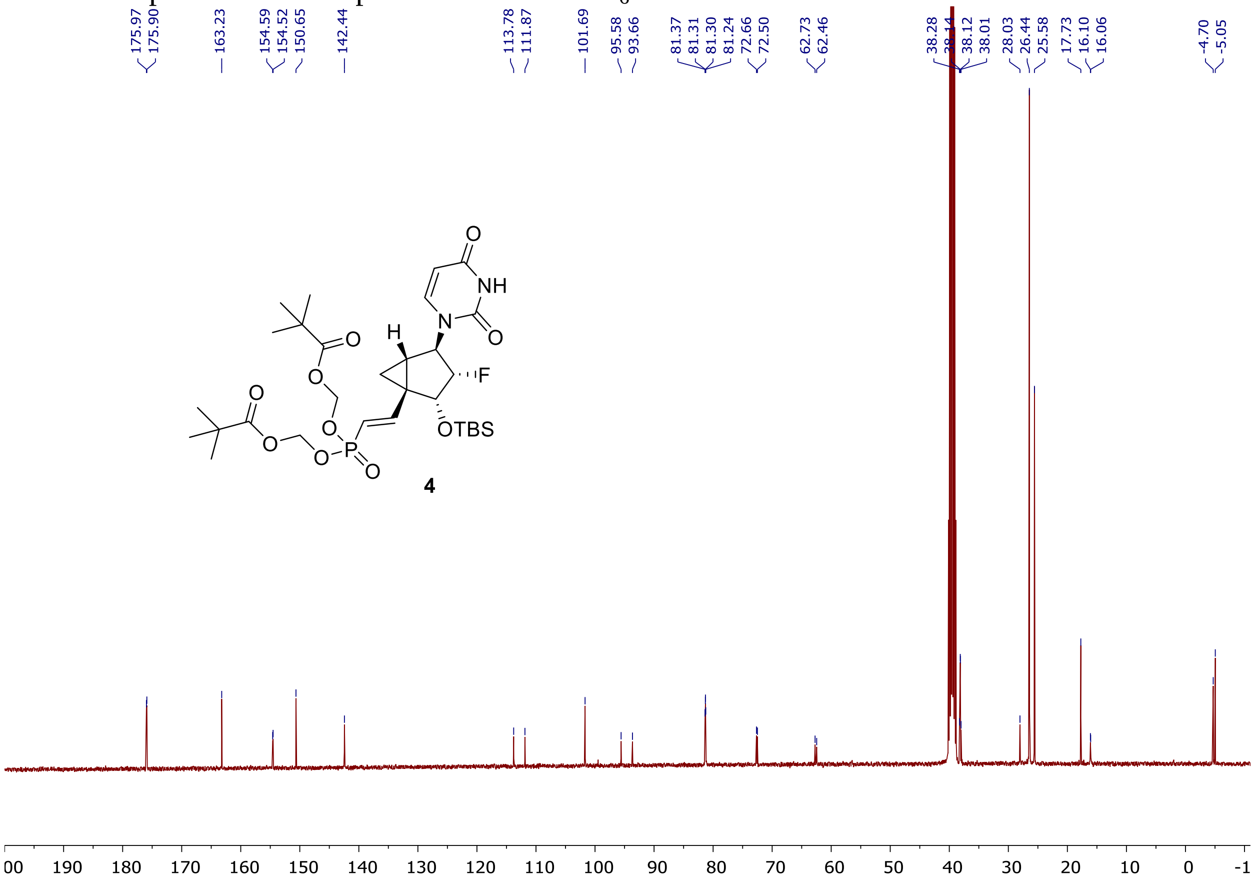

^{19}F NMR spectrum of compound **4** in DMSO-d_6

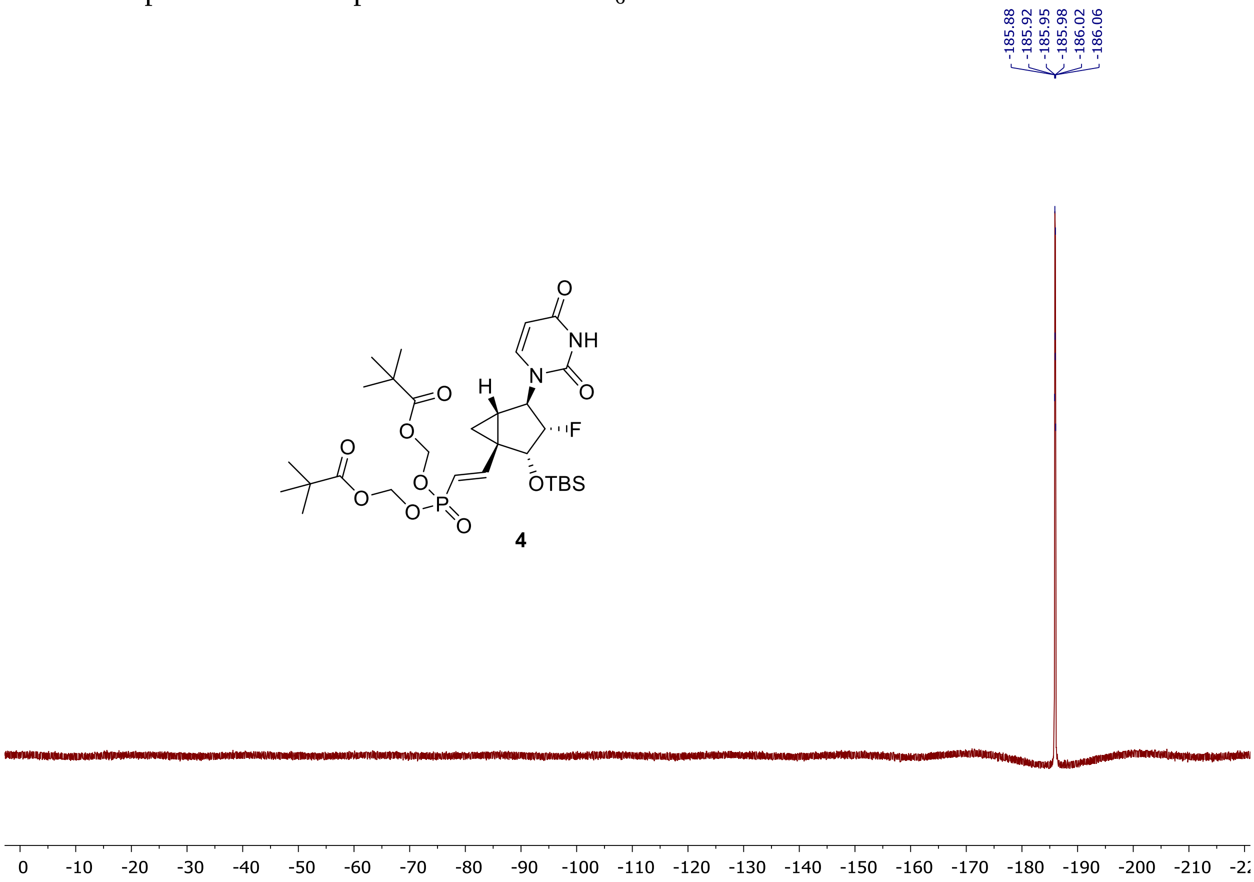

^{31}P NMR spectrum of compound **4** in DMSO-d_6

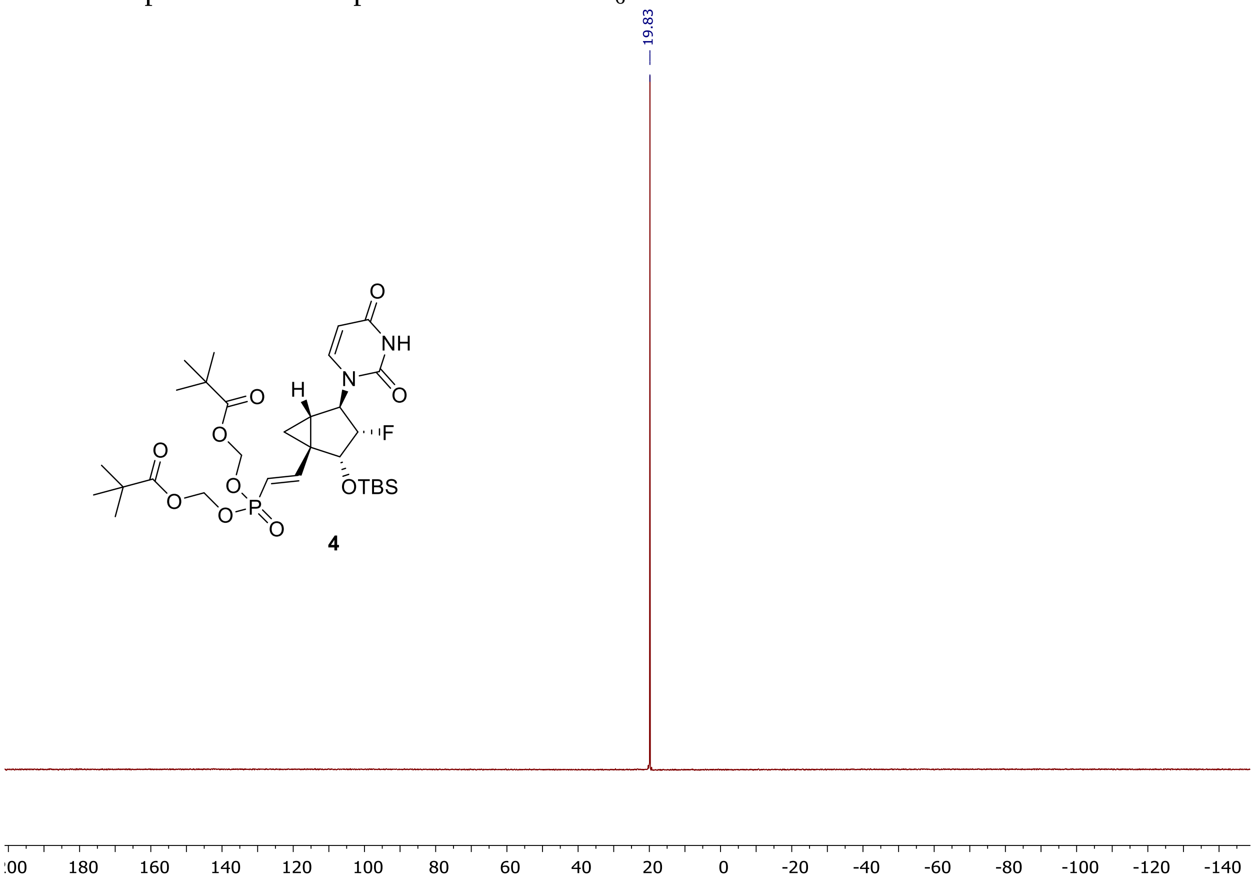

^{1}H NMR spectrum of compound **5** in DMSO-d_6

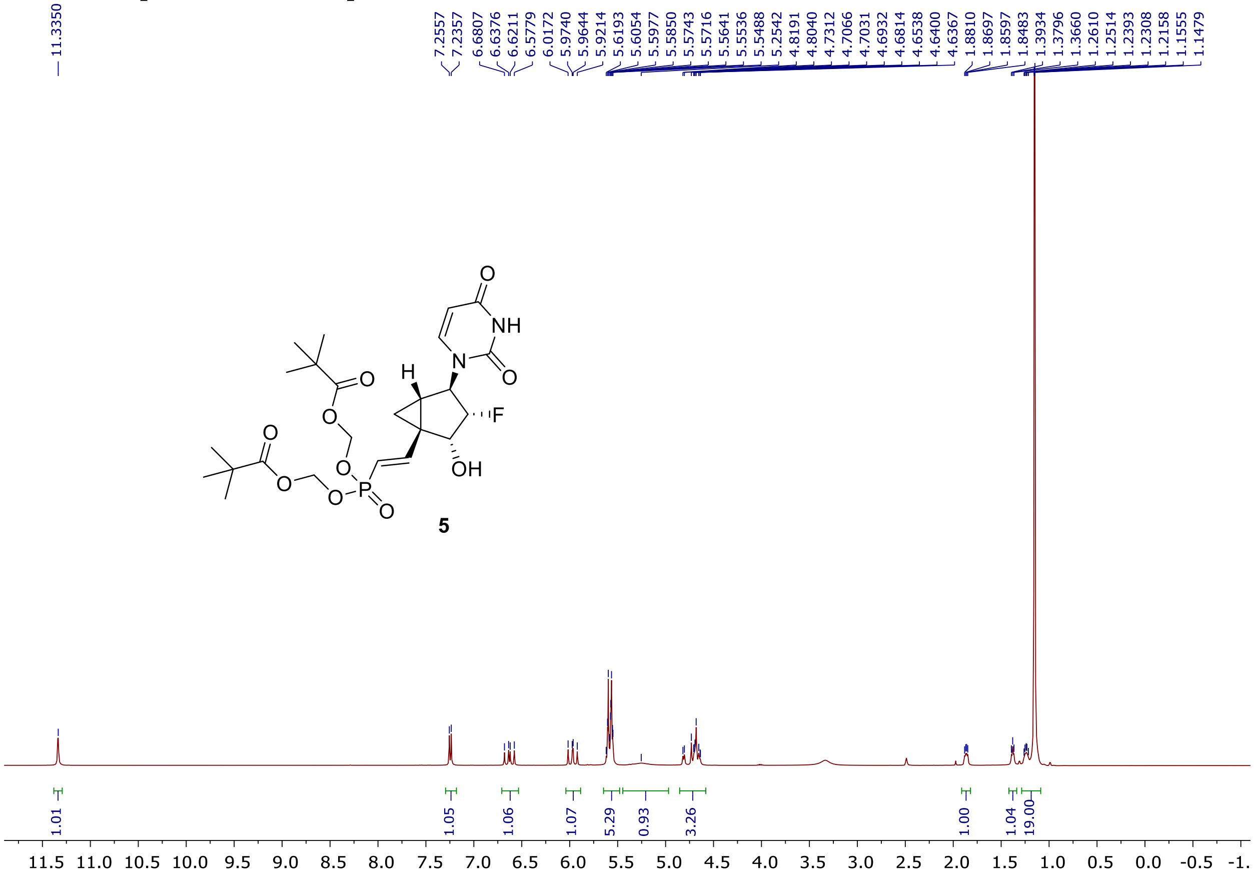

5

^{13}C NMR spectrum of compound **5** in DMSO-d_6

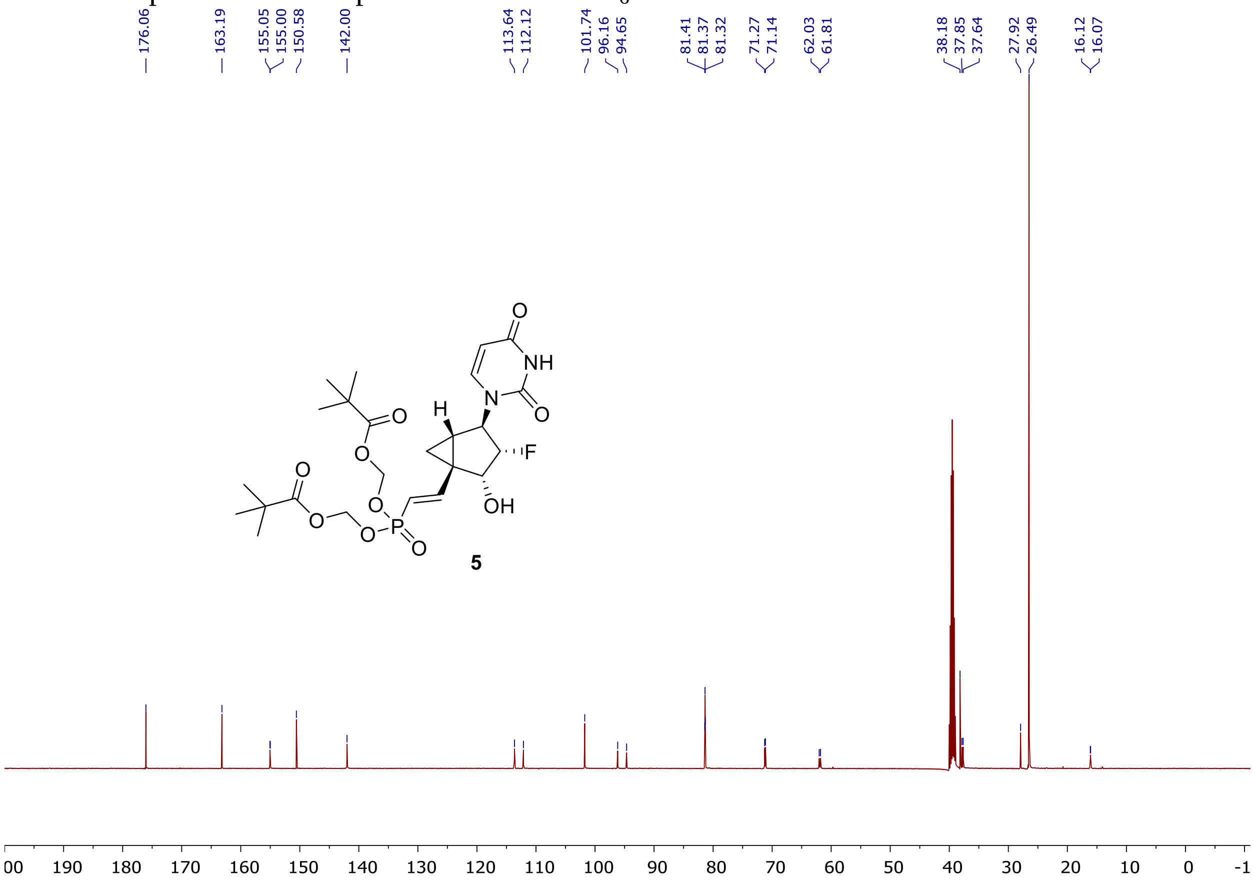

^{19}F NMR spectrum of compound **5** in DMSO-d_6

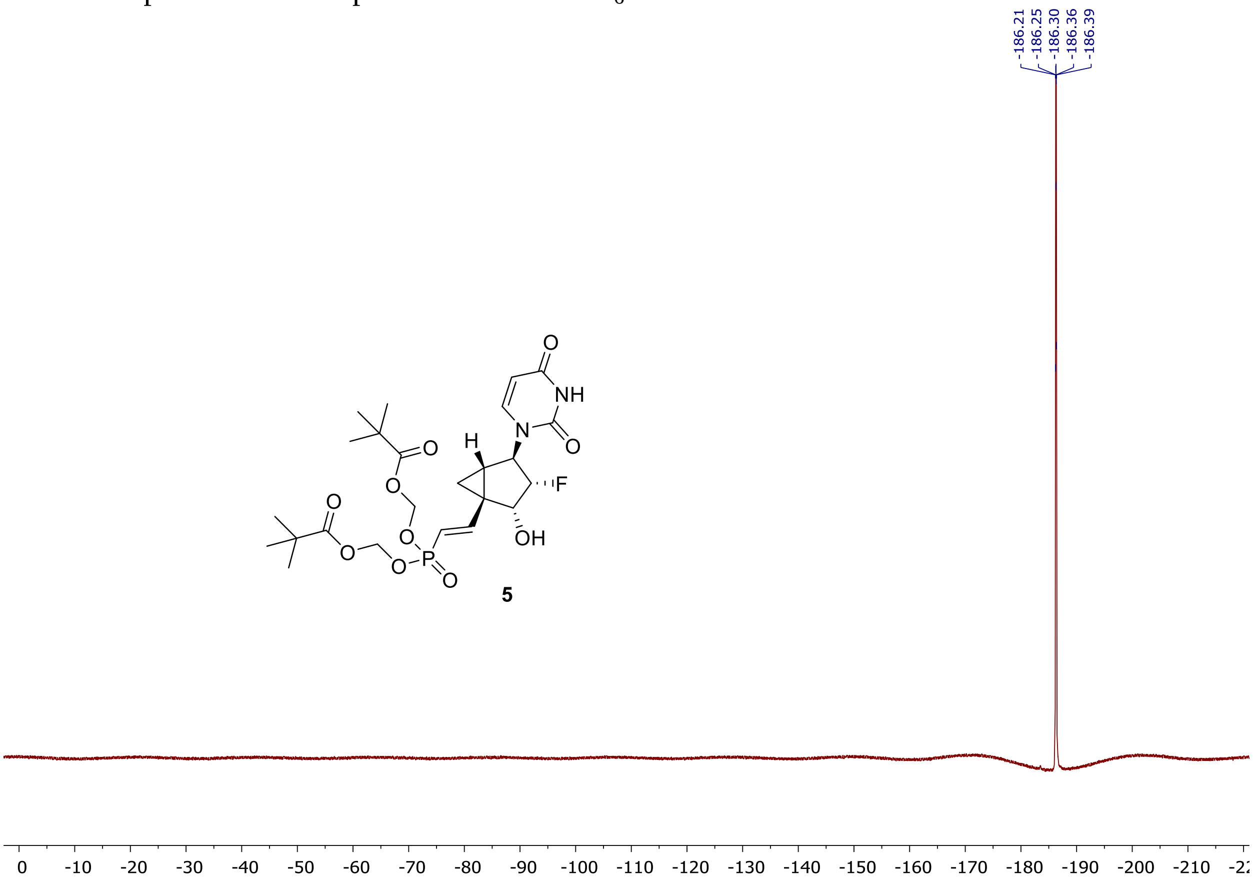

5

^{31}P NMR spectrum of compound **5** in DMSO-d_6

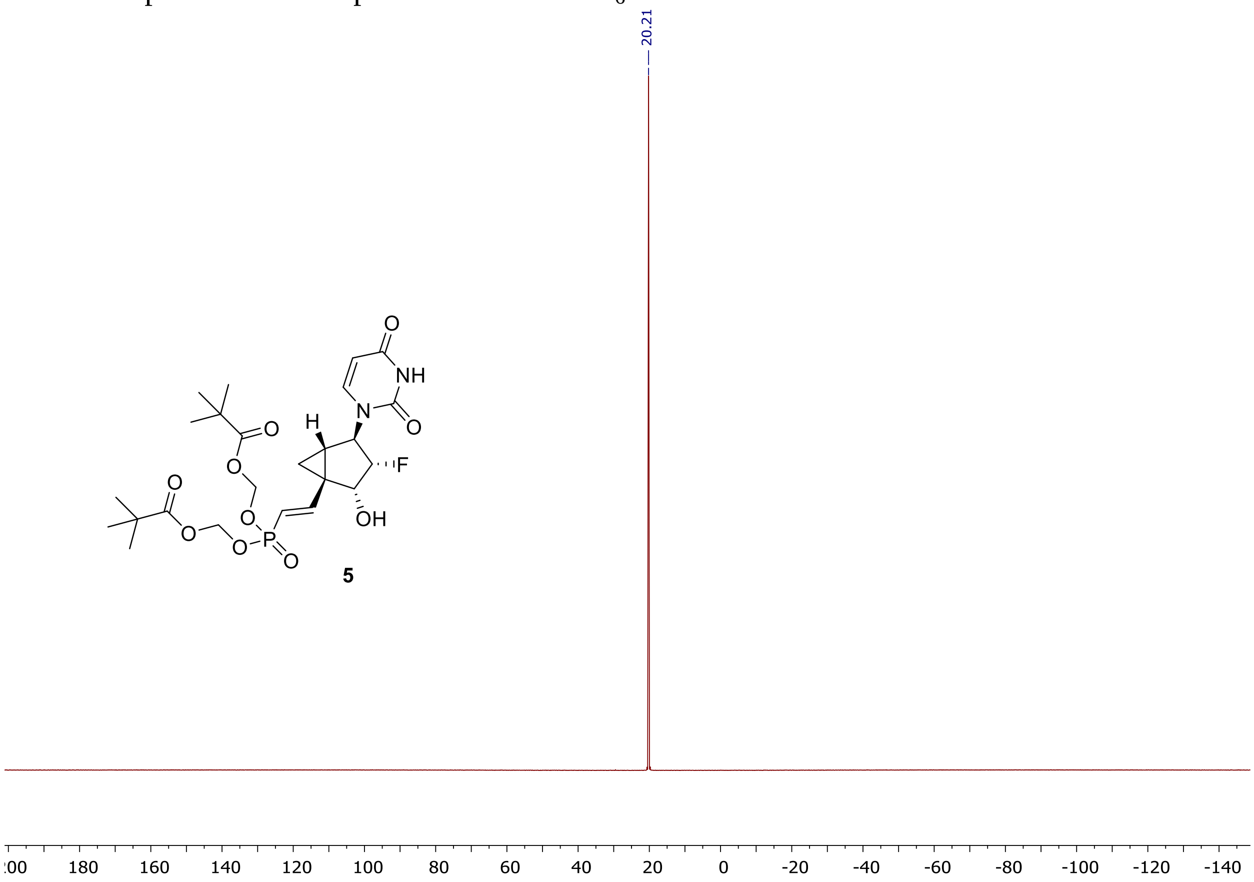

^{1}H NMR spectrum of compound **6** in CD_3CN

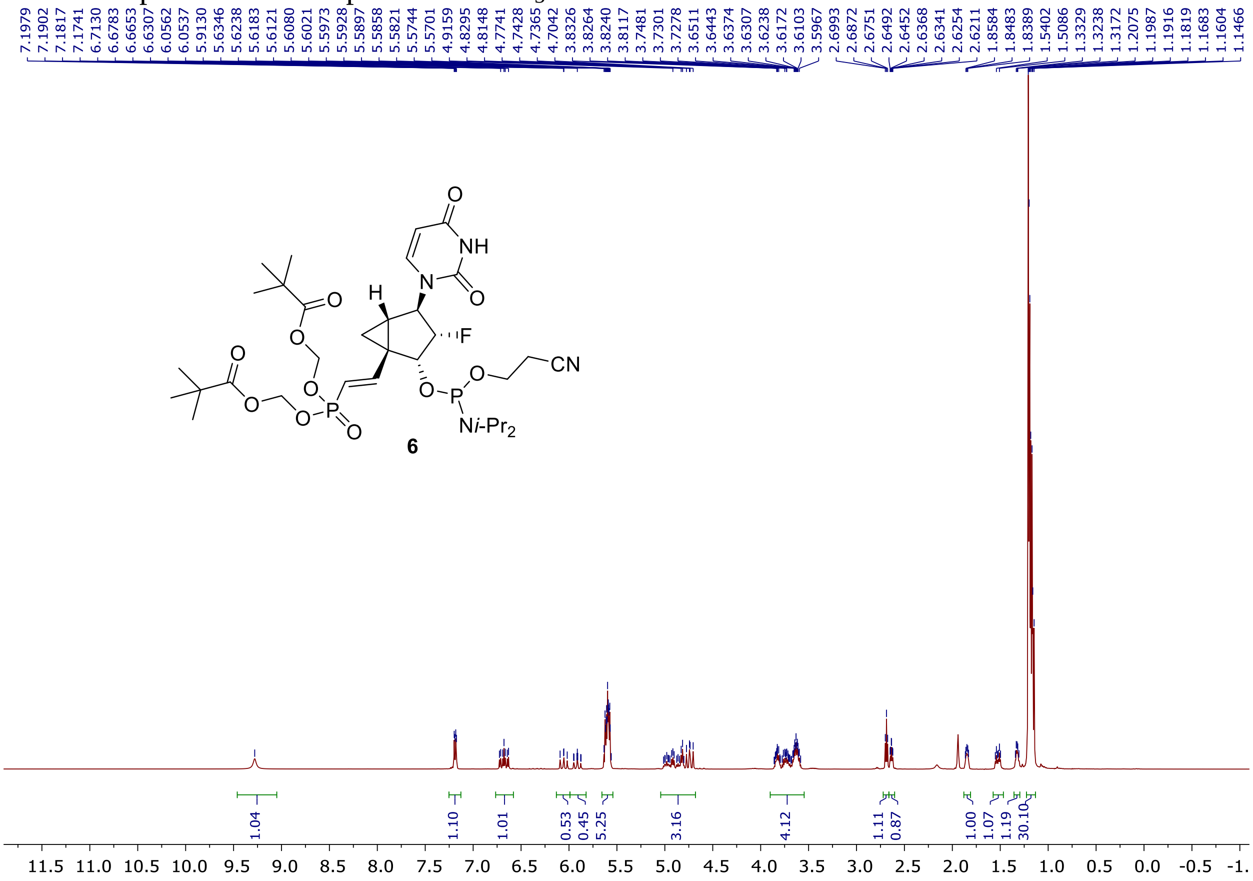

^{13}C NMR spectrum of compound **6** in CD_3CN

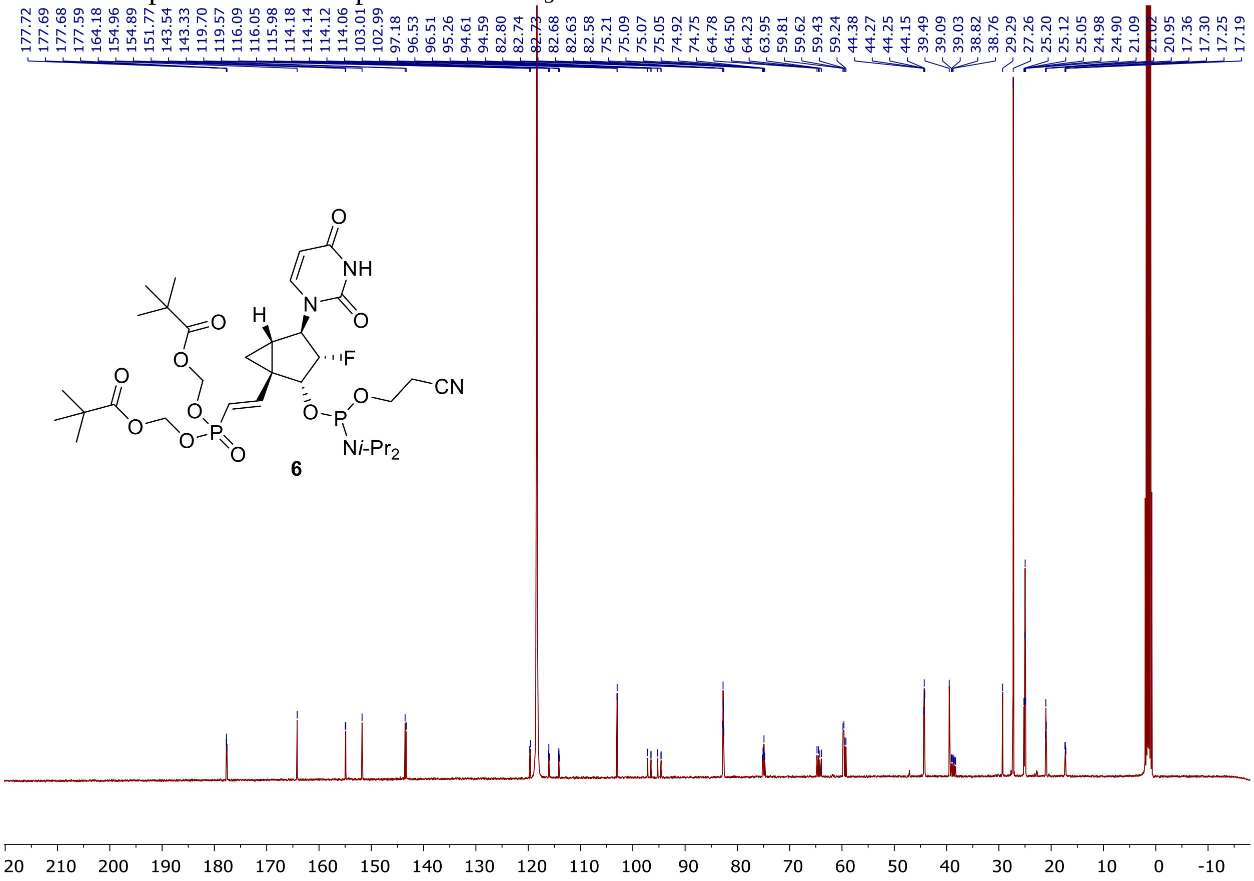

^{19}F NMR spectrum of compound **6** in CD_3CN

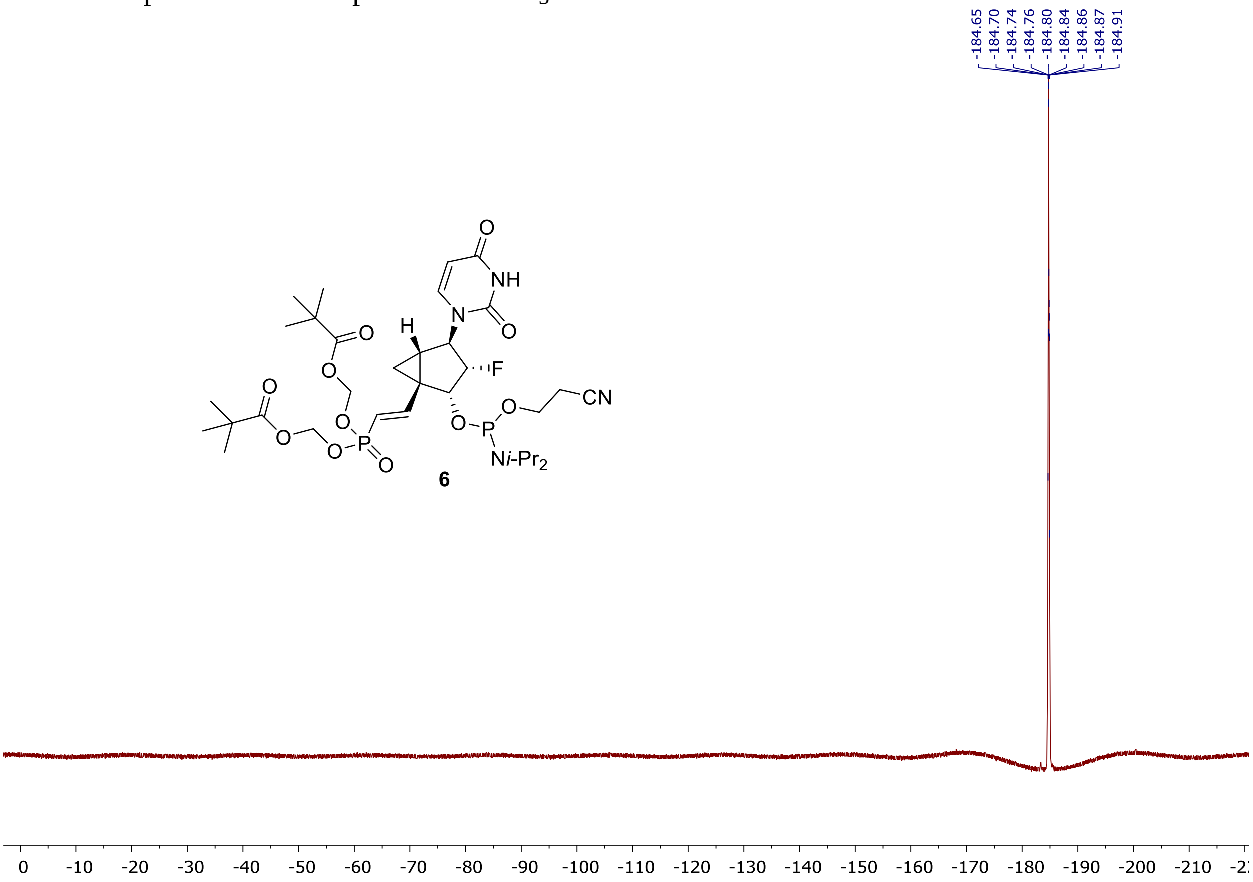

^{31}P NMR spectrum of compound **6** in CD_3CN

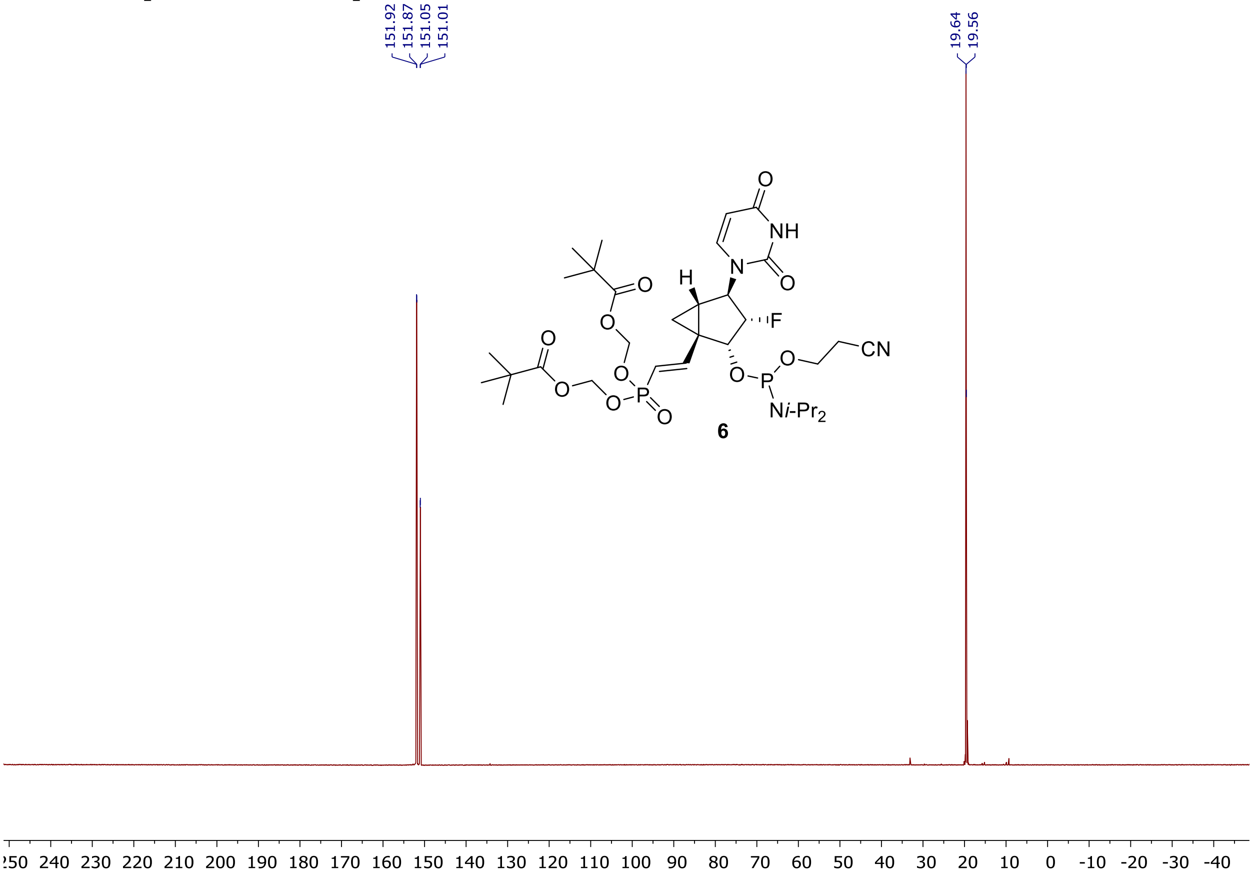

NMR spectra for 2′-F-NMC NTP **7** and **8**

^{1}H NMR spectrum of compound **7** in D_2O

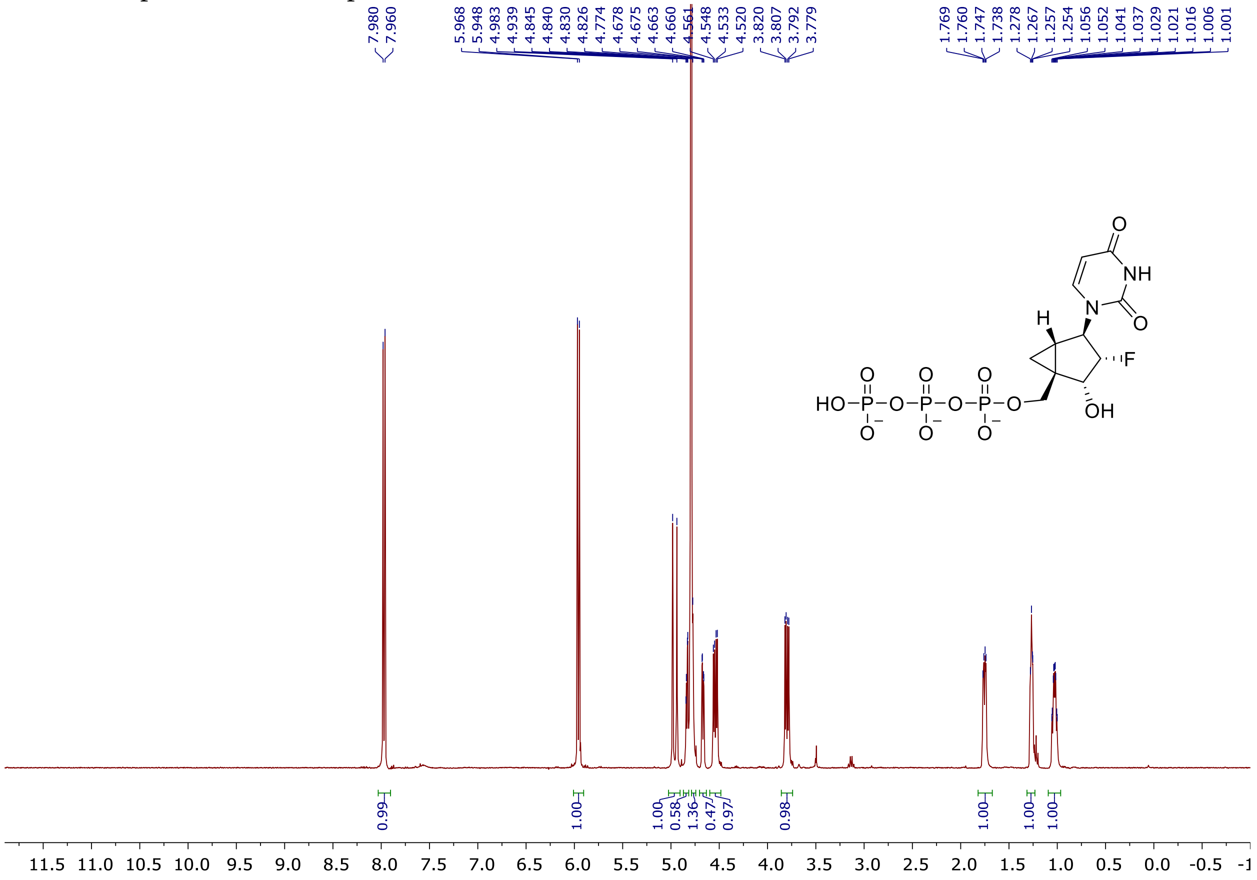

^{31}P NMR spectrum of compound **7** in D_2O

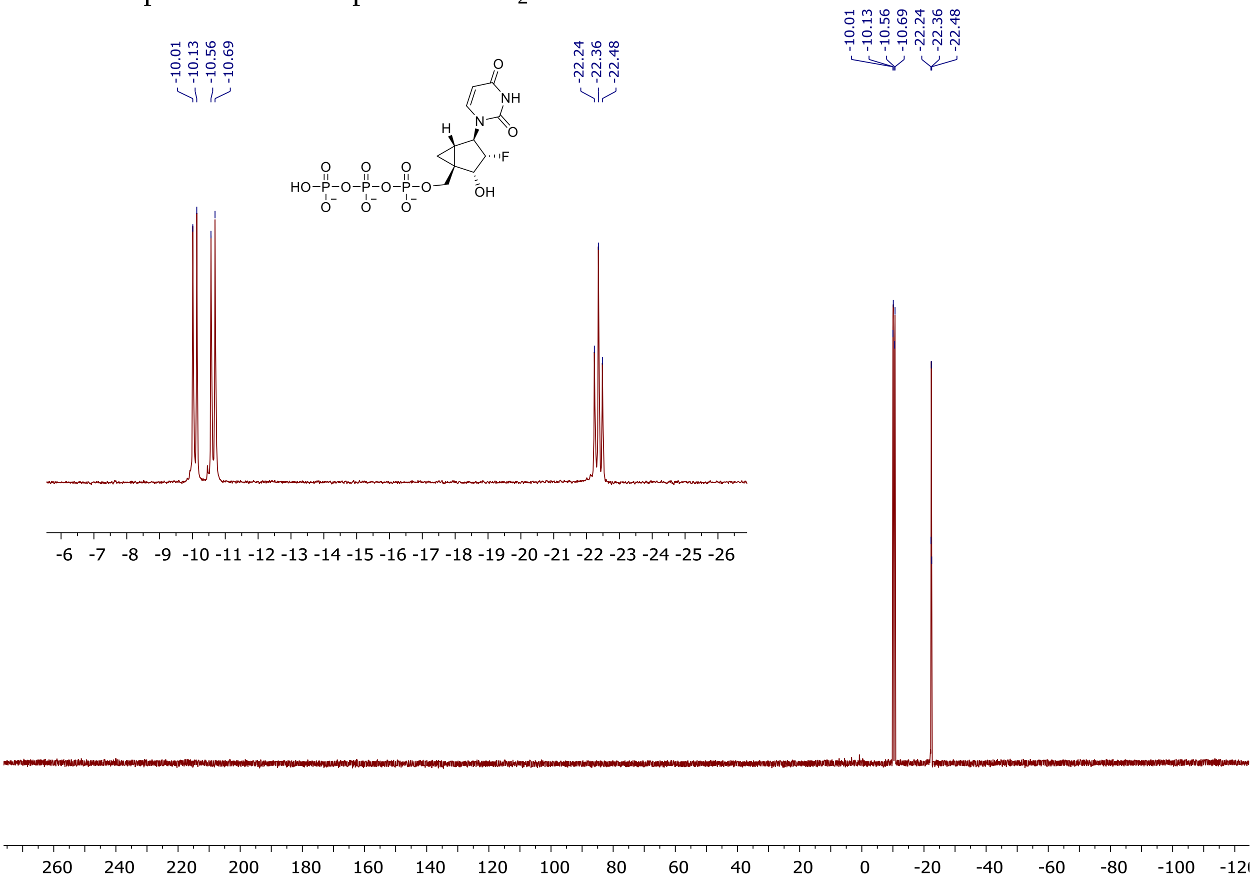

^{1}H NMR spectrum of compound **8** in D_2O

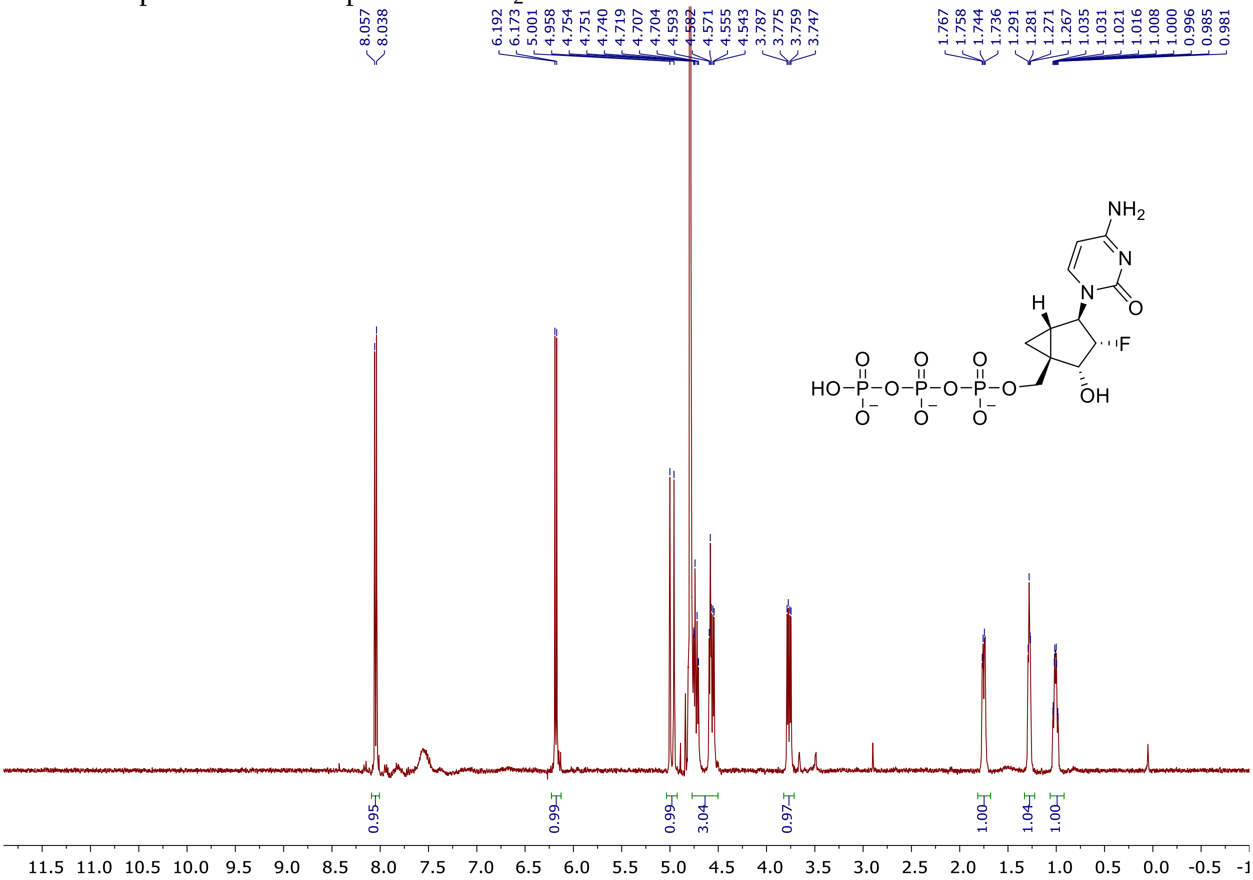

^{31}P NMR spectrum of compound **8** in D_2O

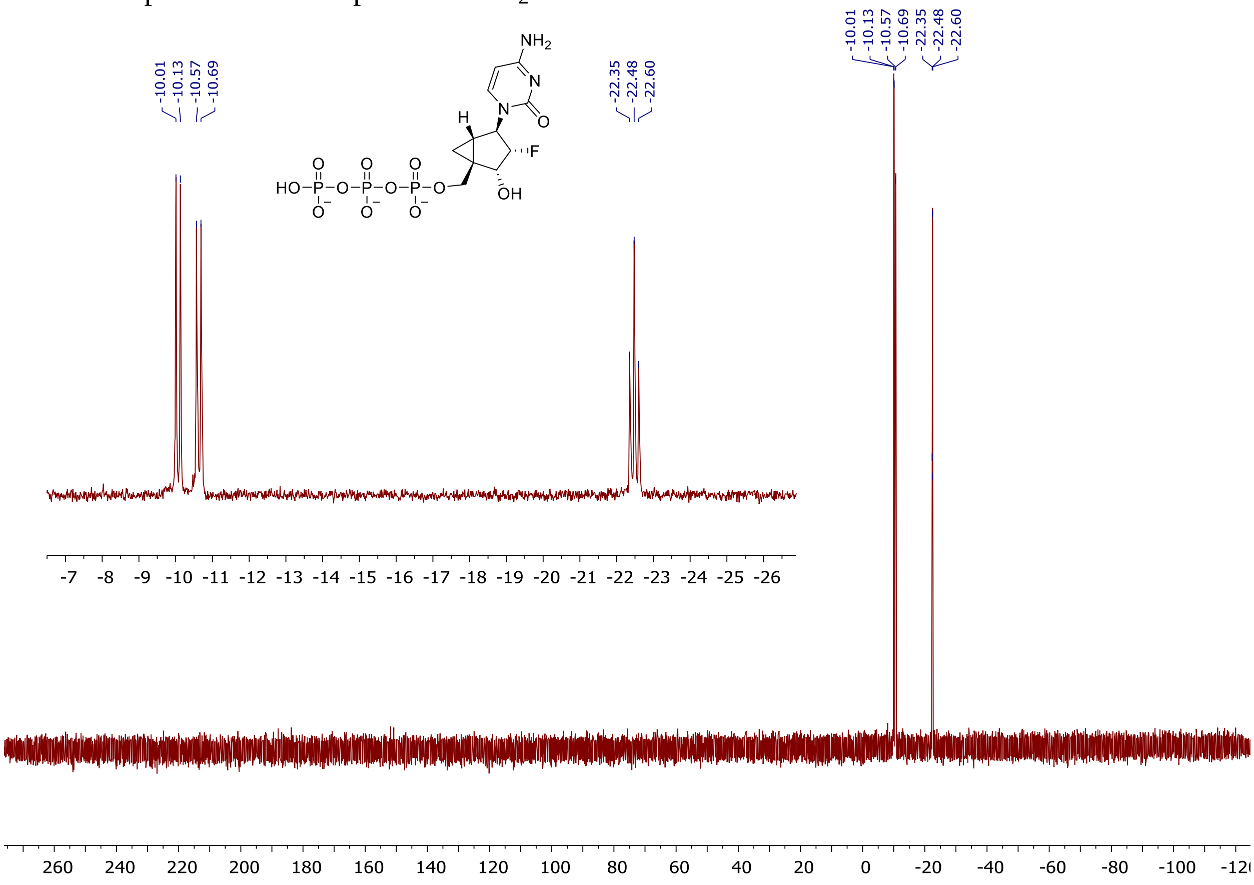

Mass components of 2′-F-NMC NTP

Mass components of compound **S7**

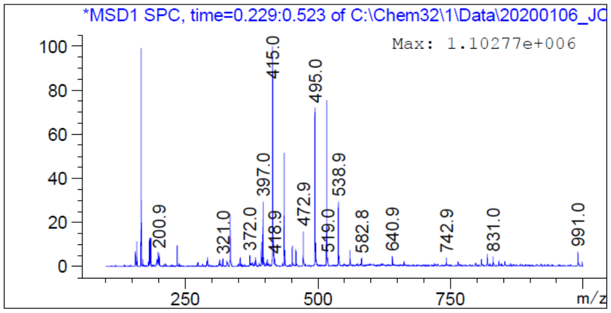

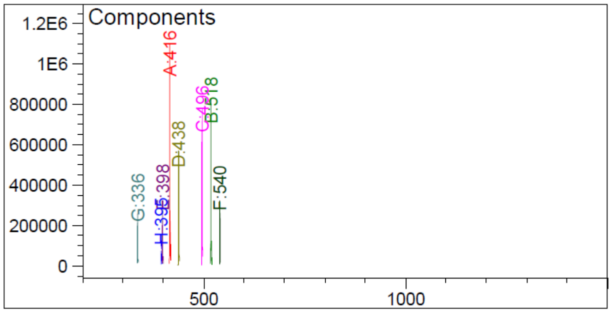

Component	Molecular Weight	Absolute Abundance	Relative Abundance
A	415.94	1102773	100.00
B	517.90	829517	75.22
C	495.93	776240	70.39
D	437.94	569419	51.64
E	397.93	323820	29.36
F	539.91	322992	29.29
G	335.97	258494	23.44
H	394.90	123588	11.21

Mass components of compound **S8**

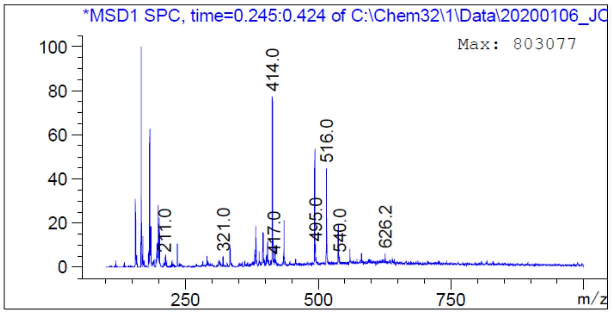

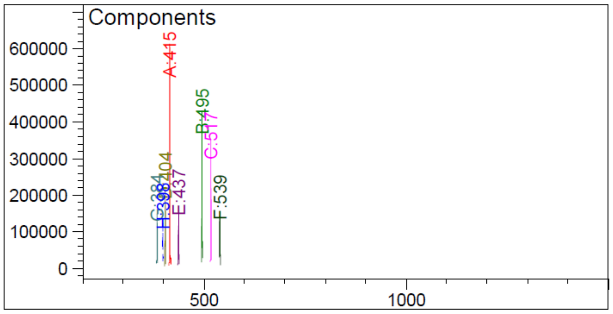

Component	Molecular Weight	Absolute Abundance	Relative Abundance
A	414.97	613794	100.00
B	494.94	430235	70.09
C	516.92	351298	57.23
D	403.88	222258	36.21
E	436.95	170608	27.80
F	538.91	156262	25.46
G	383.92	148755	24.24
H	397.92	124259	20.24

Oligonucleotide characterization

Table S1. Sequences and mass spectroscopy characterization of siRNA strands[a]

duplex	sequence (5′-3′)	strand	Mass (m/z)	
			calc.	obs.
parent[b]	A•a•CaGuGuUCUuGcUcUaUaA(L)<br>u•U•aUaGaGcAagaAcAcUgUu•u•u	sense<br>antisense	8590.2<br>7595.9	8590.2<br>7596.9
S-1(SS)	**A**•a•CaGuGuUCUuGcUcUaUaA(L)<br>u•U•aUaGaGcAagaAcAcUgUu•u•u	sense<br>antisense	8600.2<br>7595.9	8601.1<br>7597.4
S-1(OS)	**A**a•CaGuGuUCUuGcUcUaUaA(L)<br>u•U•aUaGaGcAagaAcAcUgUu•u•u	sense<br>antisense	8584.1<br>7595.9	8584.2<br>7596.6
S-1(OO)	**A**aCaGuGuUCUuGcUcUaUaA(L)<br>u•U•aUaGaGcAagaAcAcUgUu•u•u	sense<br>antisense	8568.1<br>7595.9	8569.9<br>7596.6
S-2(SS)	A•**A**•CaGuGuUCUuGcUcUaUaA(L)<br>u•U•aUaGaGcAagaAcAcUgUu•u•u	sense<br>antisense	8588.2<br>7595.9	8588.5<br>7596.6
S-2(SO)	A•**A**CaGuGuUCUuGcUcUaUaA(L)<br>u•U•aUaGaGcAagaAcAcUgUu•u•u	sense<br>antisense	8572.1<br>7595.9	8572.7<br>7596.2
S-1(S)2(S)	**A**•**A**•CaGuGuUCUuGcUcUaUaA(L)<br>u•U•aUaGaGcAagaAcAcUgUu•u•u	sense<br>antisense	8598.2<br>7595.9	8598.3<br>7597.6
S-1(O)2(O)	**AA**CaGuGuUCUuGcUcUaUaA(L)<br>u•U•aUaGaGcAagaAcAcUgUu•u•u	sense<br>antisense	8566.1<br>7595.9	8567.2<br>7597.4
S-3	A•a•**C**aGuGuUCUuGcUcUaUaA(L)<br>u•U•aUaGaGcAagaAcAcUgUu•u•u	sense<br>antisense	8600.2<br>7595.9	8601.2<br>7596.4
S-4	A•a•C**A**GuGuUCUuGcUcUaUaA(L)<br>u•U•aUaGaGcAagaAcAcUgUu•u•u	sense<br>antisense	8588.2<br>7595.9	8588.8<br>7596.5
S-5	A•a•Ca**G**uGuUCUuGcUcUaUaA(L)<br>u•U•aUaGaGcAagaAcAcUgUu•u•u	sense<br>antisense	8600.2<br>7595.9	8601.3<br>7596.3
S-6	A•a•CaG**U**GuUCUuGcUcUaUaA(L)<br>u•U•aUaGaGcAagaAcAcUgUu•u•u	sense<br>antisense	8588.2<br>7595.9	8588.8<br>7596.4
S-7	A•a•CaGu**G**uUCUuGcUcUaUaA(L)<br>u•U•aUaGaGcAagaAcAcUgUu•u•u	sense<br>antisense	8600.2<br>7595.9	8601.4<br>7597.3
S-8	A•a•CaGuG**U**UCUuGcUcUaUaA(L)<br>u•U•aUaGaGcAagaAcAcUgUu•u•u	sense<br>antisense	8588.2<br>7595.9	8588.8<br>7596.9
S-9	A•a•CaGuGu**U**CUuGcUcUaUaA(L)<br>u•U•aUaGaGcAagaAcAcUgUu•u•u	sense<br>antisense	8600.2<br>7595.9	8601.1<br>7597.6
S-10	A•a•CaGuGuU**C**UuGcUcUaUaA(L)<br>u•U•aUaGaGcAagaAcAcUgUu•u•u	sense<br>antisense	8600.2<br>7595.9	8601.1<br>7596.5
S-11	A•a•CaGuGuUC**U**uGcUcUaUaA(L)<br>u•U•aUaGaGcAagaAcAcUgUu•u•u	sense<br>antisense	8600.2<br>7595.9	8601.3<br>7597
S-12	A•a•CaGuGuUCU**U**GcUcUaUaA(L)<br>u•U•aUaGaGcAagaAcAcUgUu•u•u	sense<br>antisense	8588.2<br>7595.9	8588.7<br>7596.7

S-13	A•a•CaGuGuUCUu**G**cUcUaUaA(L)<br>u•U•aUaGaGcAagaAcAcUgUu•u•u	sense<br>antisense	8600.2<br>7595.9	8601.2<br>7597.2
S-14	A•a•CaGuGuUCUuG**C**UcUaUaA(L)<br>u•U•aUaGaGcAagaAcAcUgUu•u•u	sense<br>antisense	8588.2<br>7595.9	8588.6<br>7596.9
S-15	A•a•CaGuGuUCUuGc**U**cUaUaA(L)<br>u•U•aUaGaGcAagaAcAcUgUu•u•u	sense<br>antisense	8600.2<br>7595.9	8601.3<br>7596.9
S-16	A•a•CaGuGuUCUuGcU**C**UaUaA(L)<br>u•U•aUaGaGcAagaAcAcUgUu•u•u	sense<br>antisense	8588.2<br>7595.9	8588.5<br>7596.4
S-17	A•a•CaGuGuUCUuGcUc**U**aUaA(L)<br>u•U•aUaGaGcAagaAcAcUgUu•u•u	sense<br>antisense	8600.2<br>7595.9	8601.3<br>7596.9
S-18	A•a•CaGuGuUCUuGcUcU**A**UaA(L)<br>u•U•aUaGaGcAagaAcAcUgUu•u•u	sense<br>antisense	8588.2<br>7595.9	8588.4<br>7597
S-19	A•a•CaGuGuUCUuGcUcUa**U**aA(L)<br>u•U•aUaGaGcAagaAcAcUgUu•u•u	sense<br>antisense	8600.2<br>7595.9	8601.2<br>7597.4
S-20	A•a•CaGuGuUCUuGcUcUaU**A**A(L)<br>u•U•aUaGaGcAagaAcAcUgUu•u•u	sense<br>antisense	8588.2<br>7595.9	8588.6<br>7597
S-21	A•a•CaGuGuUCUuGcUcUaUa**A**(L)<br>u•U•aUaGaGcAagaAcAcUgUu•u•u	sense<br>antisense	8600.2<br>7595.9	8601.1<br>7597.2
AS-1(SS)	A•a•CaGuGuUCUuGcUcUaUaA(L)<br>**U**•U•aUaGaGcAagaAcAcUgUu•u•u	sense<br>antisense	8590.2<br>7593.9	8590.4<br>7594.2
AS-1(OS)	A•a•CaGuGuUCUuGcUcUaUaA(L)<br>**U**U•aUaGaGcAagaAcAcUgUu•u•u	sense<br>antisense	8590.2<br>7577.9	8590.5<br>7578.1
AS-1(OO)	A•a•CaGuGuUCUuGcUcUaUaA(L)<br>**U**UaUaGaGcAagaAcAcUgUu•u•u	sense<br>antisense	8590.2<br>7561.8	8590.6<br>7562.7
P-AS-1(SS)	A•a•CaGuGuUCUuGcUcUaUaA(L)<br>P**U**•U•aUaGaGcAagaAcAcUgUu•u•u	sense<br>antisense	8590.2<br>7672.9	8590.8<br>7674.3
P-AS-1(OS)	A•a•CaGuGuUCUuGcUcUaUaA(L)<br>P**U**U•aUaGaGcAagaAcAcUgUu•u•u	sense<br>antisense	8590.2<br>7656.9	8590.4<br>7657.8
AS-2	A•a•CaGuGuUCUuGcUcUaUaA(L)<br>u•**U**•aUaGaGcAagaAcAcUgUu•u•u	sense<br>antisense	8590.2<br>7605.9	8591.1<br>7607
AS-3	A•a•CaGuGuUCUuGcUcUaUaA(L)<br>u•U•**A**UaGaGcAagaAcAcUgUu•u•u	sense<br>antisense	8590.2<br>7593.9	8591<br>7594.4
AS-4	A•a•CaGuGuUCUuGcUcUaUaA(L)<br>u•U•a**U**aGaGcAagaAcAcUgUu•u•u	sense<br>antisense	8590.2<br>7606	8590.9<br>7607.3
AS-5	A•a•CaGuGuUCUuGcUcUaUaA(L)<br>u•U•aU**A**GaGcAagaAcAcUgUu•u•u	sense<br>antisense	8590.2<br>7593.9	8590.1<br>7594.3
AS-6	A•a•CaGuGuUCUuGcUcUaUaA(L)<br>u•U•aUa**G**aGcAagaAcAcUgUu•u•u	sense<br>antisense	8590.2<br>7606	8590.7<br>7607
AS-7	A•a•CaGuGuUCUuGcUcUaUaA(L)<br>u•U•aUaG**A**GcAagaAcAcUgUu•u•u	sense<br>antisense	8590.2<br>7593.9	8590.9<br>7594.2

AS-8	*A*•a•*C*a*G*u*G*u*UCU*u*G*c*U*c*U*a*U*a*A*(L)<br>u•*U*•a*U*a*G*a**G**c*A*aga*A*c*A*c*U*g*U*u•u•u	sense<br>antisense	8590.2<br>7606	8590.1<br>7606.8
AS-9	*A*•a•*C*a*G*u*G*u*UCU*u*G*c*U*c*U*a*U*a*A*(L)<br>u•*U*•a*U*a*G*a*G***C***A*aga*A*c*A*c*U*g*U*u•u•u	sense<br>antisense	8590.2<br>7593.9	8591.2<br>7594
AS-10	*A*•a•*C*a*G*u*G*u*UCU*u*G*c*U*c*U*a*U*a*A*(L)<br>u•*U*•a*U*a*G*a*G*c**A**aga*A*c*A*c*U*g*U*u•u•u	sense<br>antisense	8590.2<br>7606	8591<br>7607
AS-11	*A*•a•*C*a*G*u*G*u*UCU*u*G*c*U*c*U*a*U*a*A*(L)<br>u•*U*•a*U*a*G*a*G*c*A***A**ga*A*c*A*c*U*g*U*u•u•u	sense<br>antisense	8590.2<br>7593.9	8590.3<br>7594.5
AS-12	*A*•a•*C*a*G*u*G*u*UCU*u*G*c*U*c*U*a*U*a*A*(L)<br>u•*U*•a*U*a*G*a*G*c*A*a**G**a*A*c*A*c*U*g*U*u•u•u	sense<br>antisense	8590.2<br>7593.9	8590.7<br>7594.2
AS-13	*A*•a•*C*a*G*u*G*u*UCU*u*G*c*U*c*U*a*U*a*A*(L)<br>u•*U*•a*U*a*G*a*G*c*A*ag**A***A*c*A*c*U*g*U*u•u•u	sense<br>antisense	8590.2<br>7593.9	8590.8<br>7594.1
AS-14	*A*•a•*C*a*G*u*G*u*UCU*u*G*c*U*c*U*a*U*a*A*(L)<br>u•*U*•a*U*a*G*a*G*c*A*aga**A**c*A*c*U*g*U*u•u•u	sense<br>antisense	8590.2<br>7606	8590.8<br>7606.7
AS-15	*A*•a•*C*a*G*u*G*u*UCU*u*G*c*U*c*U*a*U*a*A*(L)<br>u•*U*•a*U*a*G*a*G*c*A*aga*A***C***A*c*U*g*U*u•u•u	sense<br>antisense	8590.2<br>7593.9	8589.9<br>7594.3
AS-16	*A*•a•*C*a*G*u*G*u*UCU*u*G*c*U*c*U*a*U*a*A*(L)<br>u•*U*•a*U*a*G*a*G*c*A*aga*A*c**A**c*U*g*U*u•u•u	sense<br>antisense	8590.2<br>7606	8591.1<br>7606.9
AS-17	*A*•a•*C*a*G*u*G*u*UCU*u*G*c*U*c*U*a*U*a*A*(L)<br>u•*U*•a*U*a*G*a*G*c*A*aga*A*c*A***C***U*g*U*u•u•u	sense<br>antisense	8590.2<br>7593.9	8591.5<br>7594.2
AS-18	*A*•a•*C*a*G*u*G*u*UCU*u*G*c*U*c*U*a*U*a*A*(L)<br>u•*U*•a*U*a*G*a*G*c*A*aga*A*c*A*c**U**g*U*u•u•u	sense<br>antisense	8590.2<br>7606	8591.3<br>7606.8
AS-19	*A*•a•*C*a*G*u*G*u*UCU*u*G*c*U*c*U*a*U*a*A*(L)<br>u•*U*•a*U*a*G*a*G*c*A*aga*A*c*A*c*U***G***U*u•u•u	sense<br>antisense	8590.2<br>7593.9	8590.6<br>7594.1
AS-20	*A*•a•*C*a*G*u*G*u*UCU*u*G*c*U*c*U*a*U*a*A*(L)<br>u•*U*•a*U*a*G*a*G*c*A*aga*A*c*A*c*U*g**U**u•u•u	sense<br>antisense	8590.2<br>7606	8590.3<br>7607.3
AS-21	*A*•a•*C*a*G*u*G*u*UCU*u*G*c*U*c*U*a*U*a*A*(L)<br>u•*U*•a*U*a*G*a*G*c*A*aga*A*c*A*c*U*g*U***U**•u•u	sense<br>antisense	8590.2<br>7593.9	8590.3<br>7594.1
AS-22	*A*•a•*C*a*G*u*G*u*UCU*u*G*c*U*c*U*a*U*a*A*(L)<br>u•*U*•a*U*a*G*a*G*c*A*aga*A*c*A*c*U*g*U*u•**U**•u	sense<br>antisense	8590.2<br>7593.9	8590.9<br>7594.2
AS-23	*A*•a•*C*a*G*u*G*u*UCU*u*G*c*U*c*U*a*U*a*A*(L)<br>u•*U*•a*U*a*G*a*G*c*A*aga*A*c*A*c*U*g*U*u•u•**U**	sense<br>antisense	8590.2<br>7593.9	8591<br>7594.2
AS-22(S)23	*A*•a•*C*a*G*u*G*u*UCU*u*G*c*U*c*U*a*U*a*A*(L)<br>u•*U*•a*U*a*G*a*G*c*A*aga*A*c*A*c*U*g*U*u•**U**•**U**	sense<br>antisense	8590.2<br>7591.9	8590.5<br>7592.4
AS-22(O)23	*A*•a•*C*a*G*u*G*u*UCU*u*G*c*U*c*U*a*U*a*A*(L)<br>u•*U*•a*U*a*G*a*G*c*A*aga*A*c*A*c*U*g*U*u•**UU**	sense<br>antisense	8590.2<br>7575.9	8591.2<br>7576.7
AS-1VP	*A*•a•*C*a*G*u*G*u*UCU*u*G*c*U*c*U*a*U*a*A*(L)<br>VP**U**•*U*•a*U*a*G*a*G*c*A*aga*A*c*A*c*U*g*U*u•u•u	sense<br>antisense	8590.2<br>7679.2	8590.2<br>7679.2

[a] Italicized uppercase, lowercase, and uppercase bold underlined letters represent 2′-F-RNA, 2′-OMe, and 2′-F-NMC sugar modifications, respectively, to Adenosine (A), Cytidine (C), Guanosine (G), and Uridine (U). (L) represents the tri-*N*-acetylgalactosamine (tri-GalNAc) ligand.[1] 5′-phosphate and 5′-(*E*)-vinylphosphate are indicated by the letter "P" and "VP", respectively. Phosphorothioate linkages are indicated by the "●" symbol. [b] The parent RNA duplex,[1-2] which is fully modified with 2′-OMe, 2′-F-RNA, and strategically placed PS modifications, was used for *in vitro* and *in vivo* studies as a positive control. (OS, OO, SS) refer to the first two linkages being either phosphate (O) or phosphorothioate (S), and the P in front of the code refers to the presence of a 5′ -phosphate.

Figure S1. LCMS spectrum and mass components of oligonucleotide AS-1VP

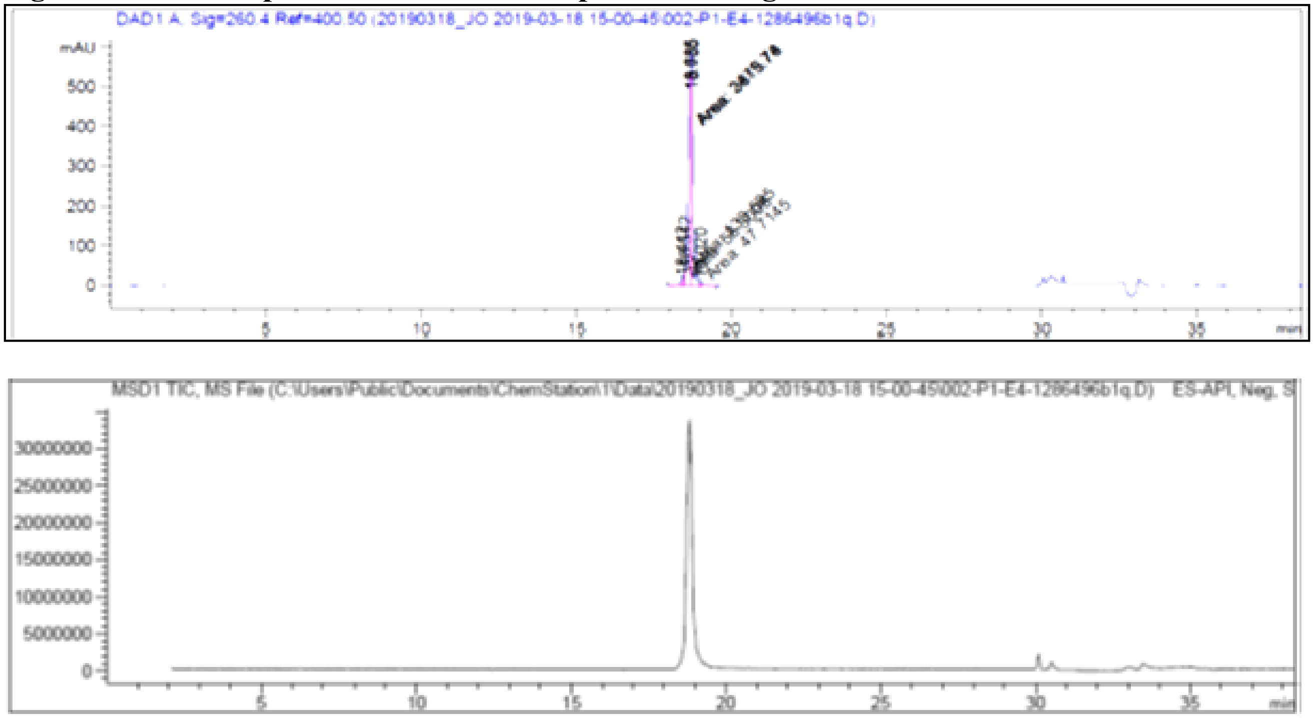

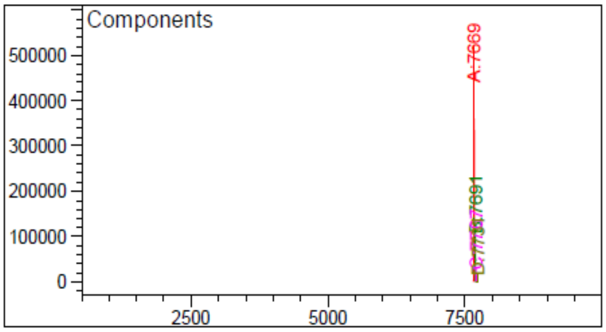

Component	Molecular Weight	Absolute Abundance	Relative Abundance
A	7669.42	520258	100.00
B	7691.35	126150	24.25
C	7707.16	30755	5.91
D	7730.93	15640	3.01

*** End of Report ***

Figure S2. Example of LC-MS spectrum of oligonucleotide S-16

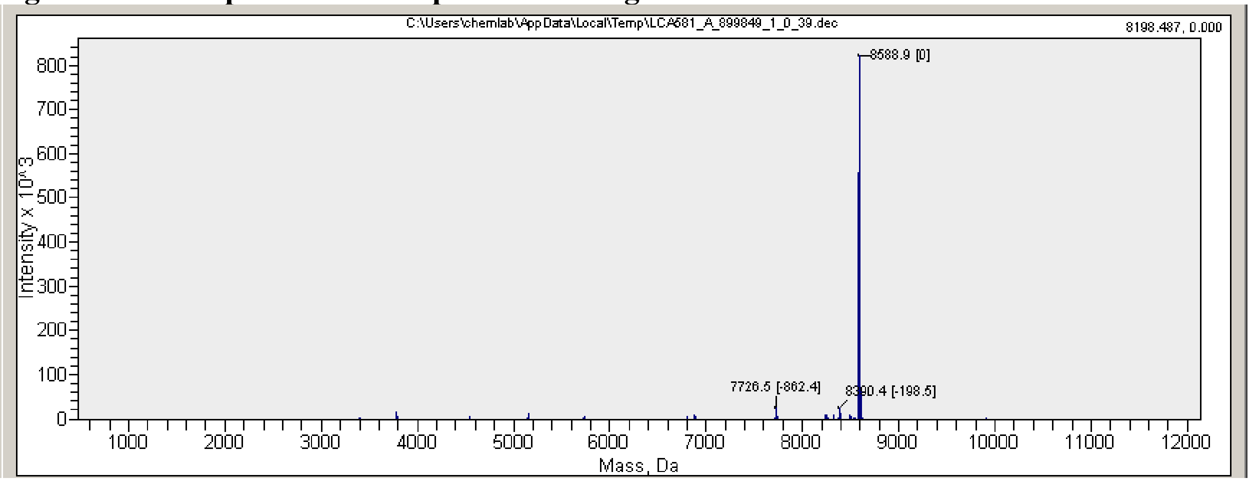

Figure S3. Example of LC-MS spectrum of siRNA duplex S-16:AS-16. The peak corresponding to S-16 is at mass 8588.7 amu; that for AS-16 is at 7598.8 amu

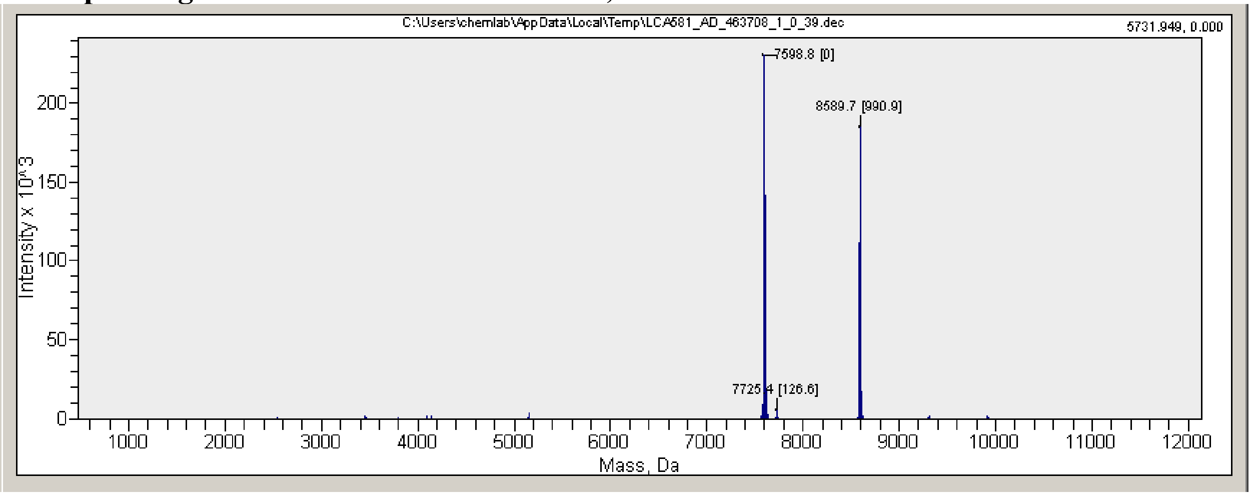

In vitro screening

Table S2. ***In vitro*** **gene silencing by duplexes modified with single 2′-F-NMC nucleotide substitution targeting mTTR mRNA**

A. Passenger Strand Walk with FNMC

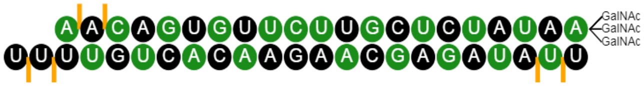

duplex[a]	IC_{50} (nM)	SD (IC_{50})
parent[b]	0.00270	0.000964
S-1(SS)	0.00290	0.001036
S-1(OS)	0.00360	0.001538
S-1(OO)	0.00400	0.001887
S-2(SS)	0.01300	0.004351
S-2(SO)	0.0193	0.012224
S-1(S)2(S)	0.00610	0.002179
S-1(O)2(O)	0.00900	0.00468
S-3	0.00460	0.001643
S-4	0.00210	0.00075
S-5	0.00670	0.002393
S-6	0.00720	0.002571
S-7	0.00330	0.001179
S-8	0.0196	0.007
S-9	0.00550	0.001964
S-10	0.01350	0.004821
S-11	0.00460	0.001643
S-12	0.00240	0.000857
S-13	0.00250	0.000893
S-14	0.00710	0.002536
S-15	0.0135	0.0087
S-16	0.00690	0.002464
S-17	0.00590	0.002107
S-18	0.00380	0.001357
S-19	0.00150	0.000536

S-20	0.00520	0.001857
S-21	0.00400	0.001429

[a] Duplex codes are referred to Table S1. [b] The parent RNA duplex,[1-2] which is fully modified with 2′-OMe, 2′-F-RNA, and strategically placed PS modifications, was used as a positive control.(Figure 1A)

B. Guide Strand Walk with FNMC

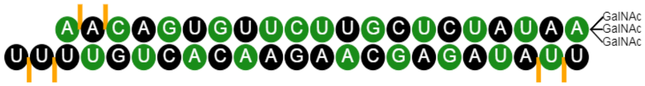

duplex[a]	IC_{50} (nM)	SD (IC_{50})
parent[b]	0.00270	0.000964
AS-1(SS)	0.0308	0.013162
AS-1(OS)	0.026	0.012264
AS-1(OO)	0.02960	0.009906
P-AS-1(SS)	0.00660	0.00418
P-AS-1(OS)	0.00240	0.000857
AS-1VP	1.014	0.0912
AS-2	0.02	0.0104
AS-3	0.00170	0.000607
AS-4	0.00320	0.001143
AS-5	0.00950	0.003393
AS-6	0.004	0.001429
AS-7	0.00440	0.001571
AS-8	0.00800	0.002857
AS-9	0.00790	0.002821
AS-10	0.00380	0.001357
AS-11	0.00430	0.001536
AS-12	0.00640	0.002286
AS-13	0.00720	0.002571
AS-14	0.0026	0.0006
AS-15	0.01110	0.003964
AS-16	0.00800	0.002857
AS-17	0.00610	0.002179
AS-18	0.00250	0.000893
AS-19	0.2267	0.0891
AS-20	0.00500	0.001786
AS-21	0.00430	0.001536

AS-22	0.0055	0.001964
AS-23	0.00850	0.003036
AS-22(S)23	0.01200	0.004286
AS-22(O)23	0.0056	0.002

[a] Duplex codes are referred to Table S1. [b] The parent RNA duplex,[1-2] which is fully modified with 2′-OMe, 2′-F-RNA, and strategically placed PS modifications, was used as a positive control. (Figure 1A)

Polymerase incorporation assay

Figure S4. Incorporation of canonical ribonucleotides and 2′-F-NMC nucleotides in the POLRMT primer extension assay. (A) Uracil containing species and (B) cytosine containing species. Ion exchange HPLC traces are represented as a function of retention time of elution (min, X-axis) and fluorescence absorbance (fluorescence units, Y-axis). All product peaks were compared to the retention time of the primer peak. Fluorescence absorbance scale is automatically normalized to the height of the major product. Labels indicate the identity of polymerase incorporation products. Representative traces of duplicate experiments are shown.

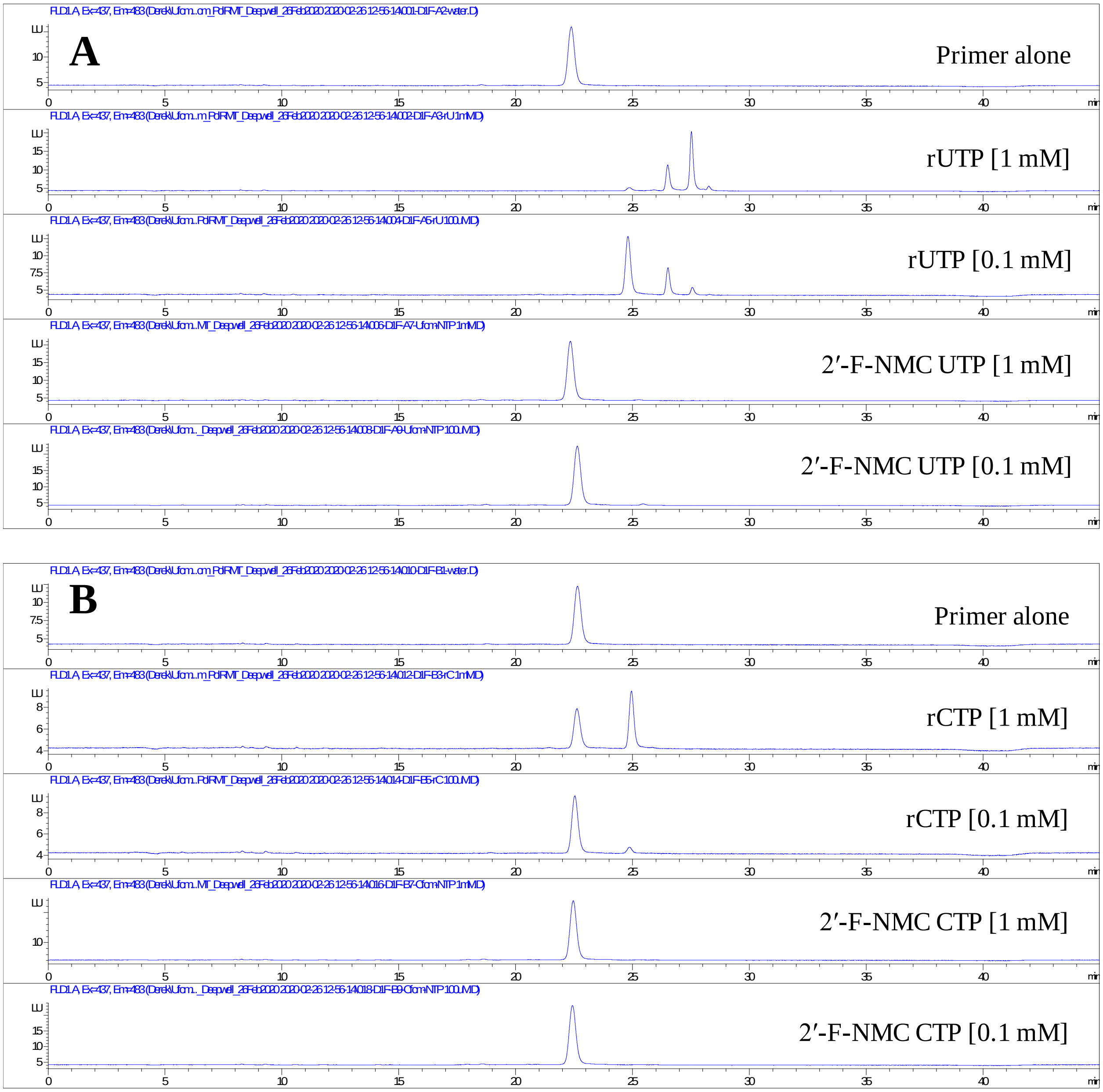

Table S3. Incorporation of 2′-F-NMC monomers in the POLRMT primer extension assay

Nucleoside triphosphate	Concentration (mM)	POLRMT extension product (%)
rUTP	1	> 98
	0.1	> 98
2′-F-dUTP*	1	31.8
2′-F-NMC UTP	1	< 1
	0.1	< 2
rCTP	1	51.8
	0.1	9.3
2′-F-dCTP*	1	58.7
2′-F-NMC CTP	1	Not detectable
	0.1	Not detectable

Calculated as 100% minus the % area of the remaining primer based on the integration of the FLD signal at λex = 436 nm and λem = 485 nm. Average value of two replicate experiments. *Note that the values for 2′-F-dNTP were obtained from previously published data.[3]

Figure S5. Incorporation of canonical 2′-deoxyribonucleotides and 2′-F-NMC nucleotides in the PolGamma primer extension assay. (A) Thymine or uracil containing species and (B) cytosine containing species. Ion exchange HPLC traces are represented as a function of retention time of elution (min, X-axis) and fluorescence absorbance (fluorescence units, Y-axis). All product peaks were compared to the retention time of the primer peak. Fluorescence absorbance scale is automatically normalized to the height of the major product. Labels indicate the identity of polymerase incorporation products. Representative traces of duplicate experiments are shown.

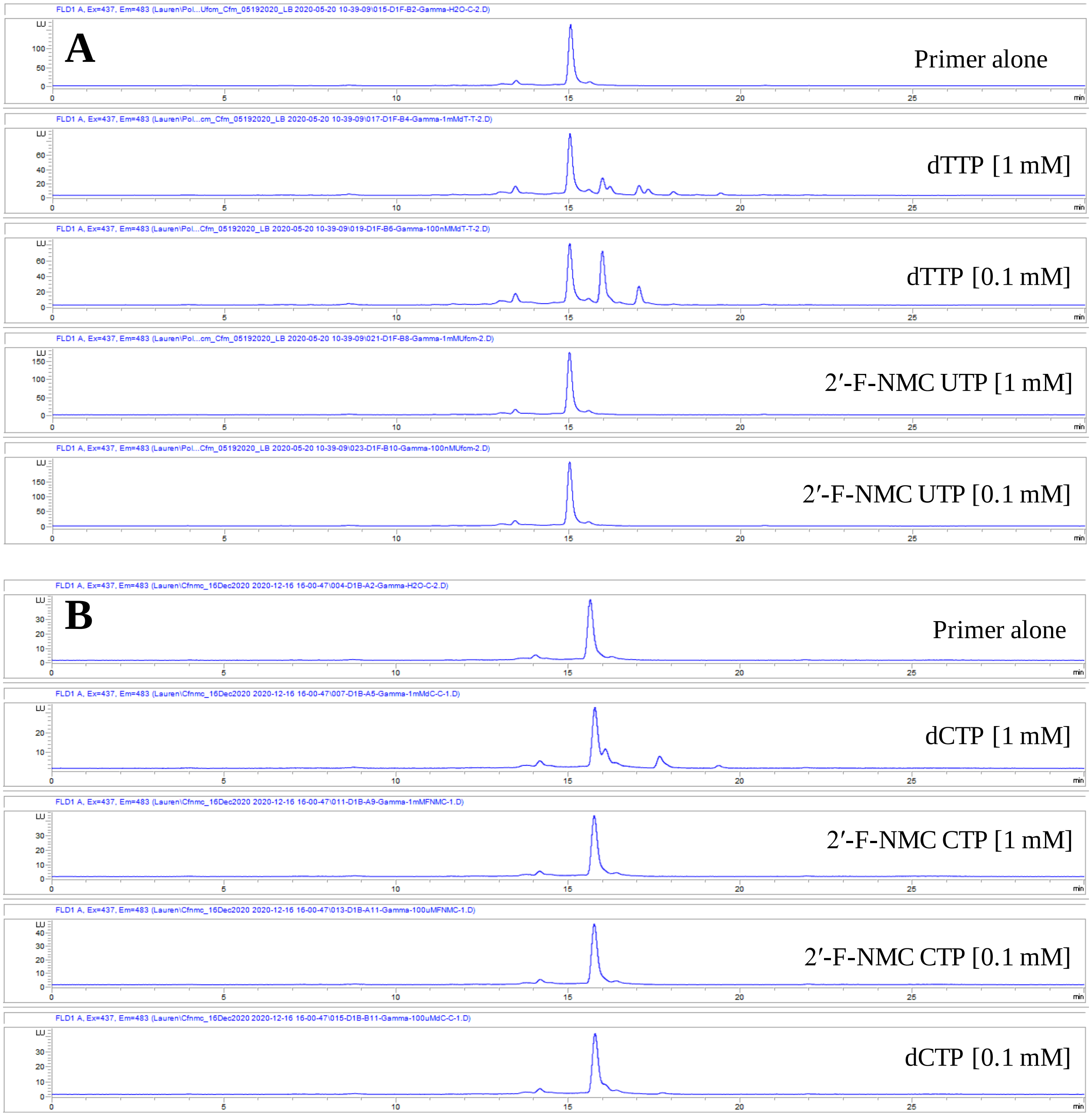

Table S4. Incorporation of 2′-F-NMC monomers in the PolGamma primer extension assay

Nucleoside triphosphate	Concentration (mM)	PolGamma extension product (%)
dTTP	1	33.7
	0.1	46.0
2′-F-dUTP*	1	87.3
2′-F-NMC UTP	1	Not detectable
	0.1	Not detectable
dCTP	1	28.3
	0.1	16.3
2′-F-dCTP*	1	94.0
2′-F-NMC CTP	1	Not detectable
	0.1	Not detectable

Calculated as 100% minus the % area of the remaining primer based on the integration of the FLD signal at λ_{ex} = 436 nm and λ_{em} = 485 nm. Average value of two replicate experiments. *Note that the values for 2′-F-dNTP were obtained from previously published data.[3]

References

1. Nair, J. K.; Willoughby, J. L. S.; Chan, A.; Charisse, K.; Alam, M. R.; Wang, Q.; Hoekstra, M.; Kandasamy, P.; Kel'in, A. V.; Milstein, S.; Taneja, N.; O'Shea, J.; Shaikh, S.; Zhang, L.; van der Sluis, R. J.; Jung, M. E.; Akinc, A.; Hutabarat, R.; Kuchimanchi, S.; Fitzgerald, K.; Zimmermann, T.; van Berkel, T. J. C.; Maier, M. A.; Rajeev, K. G.; Manoharan, M., Multivalent N-Acetylgalactosamine-Conjugated siRNA Localizes in Hepatocytes and Elicits Robust RNAi-Mediated Gene Silencing. *J. Am. Chem. Soc.* **2014,** *136* (49), 16958-16961.
2. Schlegel, M. K.; Foster, D. J.; Kel'in, A. V.; Zlatev, I.; Bisbe, A.; Jayaraman, M.; Lackey, J. G.; Rajeev, K. G.; Charisse, K.; Harp, J.; Pallan, P. S.; Maier, M. A.; Egli, M.; Manoharan, M., Chirality Dependent Potency Enhancement and Structural Impact of Glycol Nucleic Acid Modification on siRNA. *J. Am. Chem. Soc.* **2017,** *139* (25), 8537-8546.
3. Jacobson, K. A.; Tosh, D. K.; Toti, K. S.; Ciancetta, A., Polypharmacology of conformationally locked methanocarba nucleosides. *Drug Discov. Today* **2017,** *22* (12), 1782-1791.
